# SCREEN: A Graph-based Contrastive Learning Tool to Infer Catalytic Residues and Assess Enzyme Mutations

**DOI:** 10.1093/gpbjnl/qzae094

**Published:** 2024-12-26

**Authors:** Tong Pan, Yue Bi, Xiaoyu Wang, Ying Zhang, Geoffrey I Webb, Robin B Gasser, Lukasz Kurgan, Jiangning Song

**Affiliations:** Monash Biomedicine Discovery Institute and Department of Biochemistry and Molecular Biology, Monash University, Clayton, VIC 3800, Australia; Monash Biomedicine Discovery Institute-Wenzhou Medical University Alliance in Clinical and Experimental Biomedicine, Monash University, Clayton, VIC 3800, Australia; Monash Biomedicine Discovery Institute and Department of Biochemistry and Molecular Biology, Monash University, Clayton, VIC 3800, Australia; Monash Biomedicine Discovery Institute-Wenzhou Medical University Alliance in Clinical and Experimental Biomedicine, Monash University, Clayton, VIC 3800, Australia; Monash Biomedicine Discovery Institute and Department of Biochemistry and Molecular Biology, Monash University, Clayton, VIC 3800, Australia; Monash Biomedicine Discovery Institute-Wenzhou Medical University Alliance in Clinical and Experimental Biomedicine, Monash University, Clayton, VIC 3800, Australia; Monash Biomedicine Discovery Institute and Department of Biochemistry and Molecular Biology, Monash University, Clayton, VIC 3800, Australia; School of Computer Science and Engineering, Nanjing University of Science and Technology, Nanjing 210094, China; Department of Data Science and Artificial Intelligence, Monash University, Clayton, VIC 3800, Australia; Department of Veterinary Biosciences, Melbourne Veterinary School, The University of Melbourne, Parkville, VIC 3010, Australia; Department of Computer Science, Virginia Commonwealth University, Richmond, VA 23284, USA; Monash Biomedicine Discovery Institute and Department of Biochemistry and Molecular Biology, Monash University, Clayton, VIC 3800, Australia; Monash Biomedicine Discovery Institute-Wenzhou Medical University Alliance in Clinical and Experimental Biomedicine, Monash University, Clayton, VIC 3800, Australia; Key Laboratory of Clinical Laboratory Diagnosis and Translational Research of Zhejiang Province, Department of Clinical Laboratory, The First Affiliated Hospital of Wenzhou Medical University, Wenzhou 325015, China

**Keywords:** Catalytic residue, Enzyme structure, Evolutionary conservation, Graph neural network, Contrastive learning

## Abstract

The accurate identification of catalytic residues contributes to our understanding of enzyme functions in biological processes and pathways. The increasing number of protein sequences necessitates computational tools for the automated prediction of catalytic residues in enzymes. Here, we introduce SCREEN, a graph neural network for the high-throughput prediction of catalytic residues via the integration of enzyme functional and structural information. SCREEN constructs residue representations based on spatial arrangements and incorporates enzyme function priors into such representations through contrastive learning. We demonstrate that SCREEN (1) consistently outperforms currently-available predictors; (2) provides accurate results when applied to inferred enzyme structures; and (3) generalizes well to enzymes dissimilar from those in the training set. We also show that the putative catalytic residues predicted by SCREEN mimic key structural and biophysical characteristics of native catalytic residues. Moreover, using experimental datasets, we show that SCREEN’s predictions can be used to distinguish residues with a high mutation tolerance from those likely to cause functional loss when mutated, indicating that this tool might be used to infer disease-associated mutations. SCREEN is publicly available at https://github.com/BioColLab/SCREEN and https://ngdc.cncb.ac.cn/biocode/tool/7580.

## Introduction

Enzymes are critical for a wide range of diverse biochemical, molecular, and physiological processes and pathways which sustain life [1]. The extraordinary catalytic proficiency of an enzyme is often intricately orchestrated by a selected set of amino acids within its active site(s), referred to as the catalytic residues [2]. These spatially proximate catalytic residues can be engaged in critical interactions with substrate molecules, catalyzing chemical reactions and ensuring the catalytic efficiency and specificity of enzymes [3]. Catalytic residues often exhibit conservation across species, particularly those within the same taxonomic groups [4], such that mutations in catalytic sites can affect enzyme function(s), potentially inducing the onset of diseases, such as cancers and metabolic disorders [5]. For instance, mutations of catalytic residues in the tumor suppressor phosphatase and tensin homolog (PTEN) have been shown to culminate in various forms of cancers, such as glioblastoma multiforme, melanoma, and breast cancer [6]. Mutations in the catalytic sites of enzymes, such as CYP2C9, which are responsible for the biotransformation of small molecule drugs, can impact individual drug responses and potentially increase the risk of metabolic disorders [7].

The number of enzymes with detailed catalytic residue annotations available in the Mechanism and Catalytic Site Atlas (M-CSA) database [8] is substantially lower than the vast number of enzyme sequences in the UniProt database [9] and enzyme structures in repositories such as the Protein Data Bank (PDB) [10]. This gap relates primarily to the absence of high-throughput methods for identifying catalytic residues. Traditionally, the active sites in enzymes have been established using site-directed mutagenesis and biochemical assays, providing insight into the corresponding kinetic and thermodynamic parameters [11]. However, these laboratory methods are relatively low-throughput, time-consuming, and labor-intensive, thereby restricting analyses to small numbers of residues and constraining “downstream” applications such as the design of novel enzymes and inhibitors [12]. Furthermore, although M-CSA offers information on enzyme functions, its coverage of the function space is not comprehensive, particularly for oxidoreductases and translocases (with only 45.6% and 30% coverage, respectively), potentially attributable to curation backlog and/or limited functional data/information [13]. There is significant demand for an *in silico* approach for the reliable and reproducible identification of catalytic residues in enzymes from sequence and/or structure data to enable the exploration of enzyme functions and accelerate biomolecular design.

A significant effort has been directed toward developing computational methods for the identification of catalytic residues in enzymes [14]. These methods include homology-based, machine learning-based, and deep learning-based approaches. Homology-based methods annotate catalytic residues by comparing the query enzyme’s sequence or structural similarity to that of the target enzymes with experimentally validated residues. Wallace et al. firstly introduced TESS [15], a program that uses a geometric hashing algorithm to identify enzyme active sites by aligning the query enzyme to a structural template database. Mistry et al. later proposed a sequence-based method that transfers previously verified catalytic residues to other chains within the same Pfam family using a set of strict rules [16]. However, these homology-based methods are constrained by the availability of reliable templates. Compared with homology-based methods, machine learning-based and deep learning-based methods utilize the “ground truth” annotations of catalytic residues from curated public databases to train predictive models, which, in turn, can be applied to infer catalytic residues in most unknown enzymes [17]. For one of the earliest tools, Gutteridge et al. [18] trained a neural network model to identify catalytic residues using enzyme structure- and sequence-derived features as inputs. Subsequently developed predictors often employed support vector machine (SVM) models or random forest models to a collection of manually curated features [17,19–22]. Interestingly, Chea et al. [23] did not use a machine learning model but, instead, predicted catalytic residues using statistical scores calculated from a network representation of protein structure and solvent accessibility.

Structural information of the enzymes has proven highly effective in addressing diverse challenges, ranging from predicting enzyme function [24] to guiding enzyme engineering [25]. However, most existing methods for catalytic residue prediction, which rely primarily on enzyme sequences or manually curated structural features, often struggle to capture the spatial arrangements of amino acid residues, as catalytic residues tend to form spatial clusters. Moreover, the intricate chemical reactions catalyzed by enzymes are typically driven by a small subset of catalytic residues. Despite their critical role in enzyme function, current methods often fail to incorporate comprehensive enzyme functional data, thereby limiting the ability to fully explore the connections between catalytic residues, enzyme structure, and function.

We anticipate that recognizing patterns in the spatial arrangement of residues within enzyme structures can substantially enhance the performance of catalytic residue prediction tools and deepen our understanding of enzyme function through the use of modern deep neural networks. To this end, we propose here a deep learning-based solution, called SCREEN, for the accurate prediction of catalytic residues in enzymes. SCREEN employs a graph neural network that models the spatial arrangement of active sites in enzyme structures and combines data derived from enzyme structures, sequence embeddings, and evolutionary information obtained by using two complementary methods — Basic Local Alignment Search Tool (BLAST) [26] and HMMER [a sequence analysis tool using profile hidden Markov models (HMMs)] [27]. Moreover, we apply the contrastive learning framework to further enhance the predictive performance of SCREEN by incorporating enzyme functional information.

## Method

### Training and test datasets

**Figure 1:**
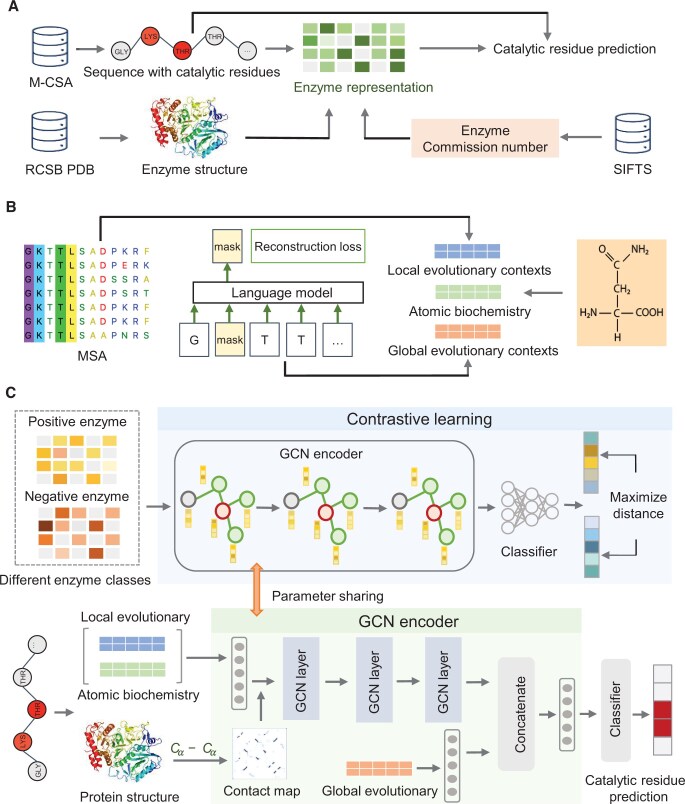
SCREEN — a predictor of catalytic residues in enzymes **A**. Data collection. We curate catalytic residue annotations from the M-CSA database, extract the corresponding enzyme structures from the RCSB PDB database, and retrieve corresponding enzyme function information using the SIFTS program [31]. **B**. Generation of inputs that include evolutionary profiles based on MSAs, sequence embeddings that leverage a large-scale protein language model, and structural characteristics derived from the atomic structures. **C**. The architecture of SCREEN’s predictive model. M-CSA, Mechanism and Catalytic Site Atlas; RCSB, Research Collaboratory for Structural Bioinformatics; PDB, Protein Data Bank; SIFTS, Structure Integration with Function, Taxonomy and Sequence; MSA, multiple sequence alignment; GCN, graph convolutional network.

We curated a dataset comprising 1055 enzymes with annotated catalytic residues, which we used to train and optimize our predictive model (**Figure 1**A). First, we collected data from M-CSA database, which contains catalytic residue annotations and details about enzymic reaction mechanisms [28]. We combined the EF family dataset that includes catalytic residue annotations for enzymes from different SCOP families [21]. We filtered the combined dataset by clustering the proteins with the CD-HIT software at 40% sequence identity [29], then randomly selected one protein from each cluster. This prevented overfitting the model into larger clusters of similar enzymes. Next, we collected enzyme three-dimensional (3D) structures from the PDB [9]. We obtained the Enzyme Commission (EC) numbers [30] using the structure Integration with Function, Taxonomy and Sequence (SIFTS) database [31]. We used the first-level EC numbers due to the relatively sparse/incomplete nature of these data at the lower levels [32]. We shuffled the dataset randomly and then divided it into training (90%) and validation (10%) subsets (Figure S1).

We acquired five widely-used test datasets to conduct a comparative evaluation of our model against existing tools. These test datasets included the EF-fold dataset, EF-superfamily dataset, and HA-superfamily dataset, representing enzymes from every SCOP fold and superfamily respectively. We also collected the PC test dataset [19], which was originally obtained from Catalytic Residue Dataset (CATRES) and represents proteins of the PIRSF protein groups [33]. Finally, we obtained the NN test dataset [18], which comprises enzymes of six main classes. Importantly, we excluded enzymes from these five test datasets from our training/validation dataset. Table S1 summarizes the training and the five test datasets.

### Overview of the SCREEN model

SCREEN is a supervised deep learner that integrates information derived from atomic structures, sequences, and evolutionary profiles. Specifically, SCREEN presents the input (enzyme structures) as graphs at the residue level, leveraging evolutionary information, sequence embeddings (generated by a modern language model), and relevant structural characteristics (*e.g.*, B-factors and solvent accessibility) (Figure 1B) [34]. We correspondingly employed a graph convolutional network (GCN) to generate propensities for catalytic residues from these inputs. The training process employed enzyme function information (EC numbers) [35] via a contrastive learning framework, utilizing the Triplet Margin Loss function to enable clustering enzymes of the same classes and separating enzymes from different classes in a latent feature space (Figure 1C). This allowed our model to develop class-specific latent feature spaces, leading to improvements in predictive performance/capacity. Moreover, we employed a dynamic training strategy, in which we initially trained the network using the contrastive learning and then applied our model to accurately identify catalytic residues (Figures S2 and S3).

### Graph-based representation of protein structure

We represented the input enzyme structure composed of *n* residues Enz = $\left\{r_{1},r_{2},\ldots ,r_{n}\right\}$ as an attributed graph encoded with evolutionary context, sequence embeddings, and relevant structural characteristics (*e.g.*, solvent accessibility and B-factors). Specifically, the graph representation G = (V,A,X) consists of 3D enzyme structure by taking the residue set V⊆Enz as graph nodes, the adjacency matrix A with n×n size that quantifies connectivity of nodes/residues, and the feature matrix $X\in R^{n\times \theta }$.

The feature matrix X = ($X_{L},X_{G},X_{A}$) covers the evolutionary, sequence, and structural features. The $X_{L}$ descriptor quantifies evolutionary conservation by utilizing two complementary tools: PSI-BLAST, which is a heuristic algorithm that relies on the dynamic programming [26], and HMMER that is based on the HMM [27]. We run PSI-BLAST on the NCBI’s non-redundant (nr) database, with three iterations and the E-value threshold of < $10^{-3}$. We normalized the output position-specific scoring matrix (PSSM) of size n×20 with the sigmoid function: $\bar{X}=\frac{1}{1+e^{-X}}$. We used HMMER with the uniclust30 database [36] and default parameters to generate the n×30 HMM matrix that we normalized to the [0, 1] range [27]. The $X_{G}$ descriptor captures sequence information computed by ProtT5 model, a deep learning language model that was pre-trained on 390 billion amino acids [37]. The enzyme sequence is encoded into residue-level feature embeddings denoted as $X_{G}\in R^{n\times h_{1}}$, where $h_{1}$ defaults to 1024. These vectors encapsulate information about individual residues that are adjacent in the sequence, and broader protein-level information. Lastly, the $X_{A}$ descriptor encompasses several key properties that are derived from the atomic-level data: atom types and atomic mass when excluding hydrogen atoms, B-factor, residue side-chain presence, the count of bonded hydrogen atoms, ring membership, van der Waals radius, and solvent accessibility. Given that residues might have different numbers of atoms, we computed the average values across all atoms, resulting in atomic descriptor $X_{A}\in R^{n\times h_{2}}$, with $h_{2}$ = 14.

### Predictive model

We designed the GCN with three convolutional layers to facilitate the propagation of feature embeddings for residues that share spatial proximity.

For a given graph defined by the adjacency matrix $A\in {\left\{0,1\right\}}^{n\times n}$ and the feature matrix X= ($X_{L},X_{G},X_{A}$), our model produces residue-level representations $H^{\left(i\right)}\in R^{n\times d_{i}}$, where $d_{i}$ represents the embedding dimension for the i-th convolutional layer.


(1) $$\begin{array}{c} H^{\left(i\right)}=\mathrm{GCN}\left(A,\left[X_{L},X_{A}\right]\right) \end{array}$$

We refined residue representations through the process of neighbor aggregations as follows:


(2) $$\begin{array}{c} H^{\left(i\right)}=\mathrm{ReLU}\left({\tilde{D}}^{-\frac{1}{2}}\left(A+I_{n}\right){\tilde{D}}^{-\frac{1}{2}}H^{\left(i-1\right)}W^{\left(i\right)}\right) \end{array}$$

(3) $$\begin{array}{c} H^{\left(0\right)}=\left[X_{L},X_{A}\right] \end{array}$$

where $I_{n}\in R^{n\times n}$ is the identity matrix, $\tilde{D}\in R^{n\times n}$ is the diagonal degree matrix with entries${\;D}_{\mathrm{ii}}$=$\sum_{j}{\left(A+I_{n}\right)}_{\mathrm{ij}}$, $W^{\left(1\right)}\in R^{\theta \times d_{i}}$ is the trainable weight matrix for the i-th convolutional layer, ReLU denotes the Rectified Linear Unit activation function, and [] denotes the concatenation operation. The above architecture generates graph representation $X_{E}\in R^{n\times d}$, where *d* = 512 (Figure S4), which we combined using multilayer perception (MLP) network as follows:


(4) $$X_{E}=\mathrm{ReLU}\left(\mathrm{MLP}\left(\left[\mathrm{ReLU}\left(\mathrm{MLP}\left(\left[H^{1},H^{2},H^{3}\right]\right)\right),\mathrm{ReLU}\left(\mathrm{MLP}\left(X_{G}\right)\right)\right]\right)\right)$$

We employed three fully connected layers in the MLP network to reduce the feature space to the final output vector $Y\in R^{n\times 2}$ that gives numeric propensities for putative catalytic residues.

### Contrastive learning

We used contrastive learning with Triplet Margin Loss to craft enzyme representations that improve the catalytic residue predictions. Using the graph representation $X_{E}\in R^{n\times d}$, we employed average aggregation across residues, generating a fixed-sized sequence representation vector $z\in R^{1\times h_{3}}$, with $h_{3}$ set to 1024. During training in each epoch, we iteratively refined every sequence representation vector and computed enzyme class cluster centers. When training with a query enzyme $z_{a}$, we selected the enzyme cluster center embedding from the same enzyme class as the positive sample $z_{p}$, and randomly sampled another cluster center from a different enzyme class as the negative sample $z_{n}$, which resulted in the following Triplet Margin Loss function:


(5) $$\begin{array}{c} L^{\mathrm{TM}}=∥z_{a}-\;z_{p}{∥}_{2}-∥z_{a}-\;z_{n}{∥}_{2}+\alpha \end{array}$$

where we set the margin *α* to the default value of 1. This loss function minimizes the Euclidean distance between enzyme representations belonging to the same main enzyme class while maximizing the distance between those form different main enzyme classes. We implemented a dynamic training strategy, where we performed contrastive learning for enzyme classification during early training epochs, and gradually shifted toward the default training that converges to produce accurate propensities for catalytic residues.

### The multiplexed assays of variant effects data analyses

We gathered the multiplexed assays of variant effects (MAVE) measurements for four enzymes, which provided insight into the impact of a broad collection of substitutions on both enzyme function and abundance [6,38]. We categorized the missense variants of PTEN into two main groups: functional or inactive, regardless of their effects on abundance. The classification thresholds for the scores generated by each MAVE were guided by an established methodology [39]. Specifically, we used a minimal number of Gaussians (three) to ensure a reliable fit to the variant score distributions, and the intersection point between the first and last Gaussians served as the classification cut-off. Adopting this binary classification approach allowed us to categorize variants into two classes: (1) wild type-like (WTL), variants characterized by high activity; (2) functional loss (FL), variants assigned that exhibit low activity.

## Results

### SCREEN accurately predicts catalytic residues

**Figure 2:**
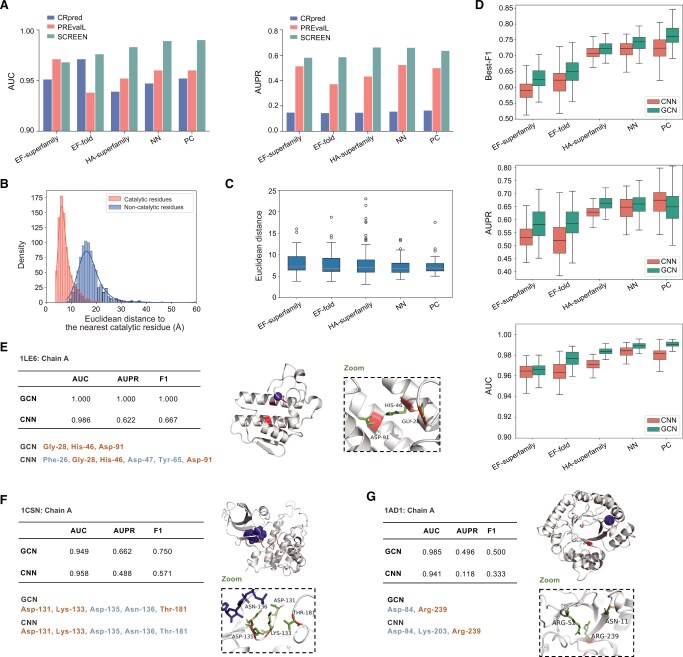
Predictive performance of SCREEN **A**. Performance comparison of SCREEN with the sequence-based CRpred and structure-based PREvaIL on the five test datasets. **B**. The distribution of the Euclidean distances to the nearest catalytic residues for the residues in enzymes from the M-CSA database [13]. **C**. The distribution of the Euclidean distances to the nearest catalytic residues for the catalytic residues predicted by SCREEN. For boxplot, the center line represents the median value, and the top and bottom edges are the first and third quartiles, respectively. **D**. Performance comparison of the structure-based SCREEN model (GCN encoder) with a sequence-based baseline model (CNN encoder) on the five test datasets. **E**.–**G**. Three examples, ranked approximately at 10% (E), 50% (F), and 90% (G) based on the Best-F1 scores for catalytic residues predicted by SCREEN. The structure-based SCREEN (GCN encoder) was compared with a sequence-based baseline model (CNN encoder). The native catalytic residues are in green in a zoomed figure. The correctly identified catalytic residues by SCREEN and the baseline model (CNN encoder) are marked in orange, while misidentified residues are in gray. AUC, area under the receiver operating curve; AUPR, area under the precision-recall curve; CNN, convolutional neural network.

We collected five commonly-used test datasets to comparatively assess SCREEN against eight current solutions. These test datasets included the EF-superfamily and EF-fold datasets [21], the HA-superfamily dataset [23], the NN dataset [18], and the PC dataset [19]. We compared the results from SCREEN with those of a conventional sequence-based method, CRpred [17], and six tools that employed different predictive models based on enzyme structures. These tools included a neural network-based approach [18], three SVM-based methods [19], a random forest-based PREvaIL [22], and a statistical approach [23]. We compared the precision, recall, and F1 score to evaluate the predictions of catalytic residues, referencing the reported performance from the original paper (**Table 1**; Figure S5). We also evaluated the recently proposed graph-based method, AEGAN [40], by retraining and testing it on the same training and test datasets, with the hyperparameter for negative sample size set to 20. We showed that SCREEN consistently outperformed the eight tools for the five test datasets. Compared to these methods, SCREEN achieved a higher F1 score across all five test datasets. The high F1 scores achieved by SCREEN were coupled with balanced and high values of precision and recall that ranged between 61.0 and 69.3 and between 61.2 and 82.0, respectively. We also quantified and compared two other popular metrics, the area under the receiver operating curve (AUC) and the area under the precision-recall curve (AUPR). **Figure 2**A reveals that SCREEN achieved substantially higher AUC and AUPR scores when compared with the latest structure-based (PREvaIL) and the sequence-based (CRpred) tools, except for the EF-superfamily and EF-fold datasets, where AUC and AUPR values were comparable.

**Table 1:** Performance comparison of SCREEN with existing methods for catalytic residue prediction

Method	Measurement (%)	EF-superfamily dataset	EF-fold dataset	HA-superfamily dataset	NN dataset	PC dataset
Methods from Youn et al. [21], Chea et al. [23], Gutteridge et al. [18], and Petrova and Wu [19]	Precision (Recall)	${16.9\;(53.9)}^{a}$	${17.1\;(51.1)}^{a}$	${16.5\;(29.3)}^{b}$	${56.0\;(14.0)}^{c}$	${7.0\;(90.0)}^{d}$
	F1 score	25.7	25.6	21.1	22.4	13.0
CRpred [17]	Precision (Recall)	15.9 (52.1)	16.1 (48.0)	24.7 (49.7)	65.9 (18.0)	5.6 (84.5)
	F1 score	24.4	24.1	33.0	28.3	10.5
CRHunter [20]	Precision (Recall)	21.5 (68.7)	21.0 (62.7)	33.2 (69.6)	76.4 (24.0)	7.6 (92.1)
	F1 score	32.8	31.5	45.0	36.5	14.0
PREvaIL [22]	Precision (Recall)	17.0 (59.4)	17.0 (56.5)	17.0 (57.9)	58.9 (17.0)	17.0 (58.1)
	F1 score	26.4	26.1	26.3	26.4	26.3
AEGAN [40]	Precision (Recall)	31.7 (85.7)	31.0 (83.7)	29.8 (85.7)	29.6 (83.9)	28.6 (86.6)
	F1 score	45.9	44.6	43.6	43.3	41.3
SCREEN (this study)	Precision (Recall)	61.9 (61.2)	61.0 (68.4)	69.3 (74.8)	68.5 (79.9)	67.6 (82.0)
	F1 score	61.5	64.5	72.0	73.8	74.1

*Note*: *^a^* Model performance on the EF-superfamily and EF-fold datasets by Youn et al. [21]; ^*b*^ Model performance on the HA-superfamily dataset by Chea et al. [23]; *^c^* Model performance on the NN dataset using the structure-based method without spatial clustering by Gutteridge et al. [18]; ^*d*^ Model performance on the PC dataset by Petrova and Wu [19].

Using SCREEN, we also measured Best-F1 scores and AUPR values for specific enzyme types (Figure S6). Particularly, for hydrolases, which represent the largest portion of the training dataset (313/1055, 29.7%), SCREEN obtained notable consistency across the five test datasets, with Best-F1 scores ranging from 0.63 to 0.78 and AUPR values ranging from 0.56 to 0.68. For the isomerase data, the predictive performance was particularly high, with Best-F1 score exceeding 0.74 and AUPR surpassing 0.80, even though these enzymes represent only a small portion of the training dataset (87/1055, 8.2%).

### The use of enzyme structure information in SCREEN markedly improves the catalytic residue prediction

The catalytic residues typically tend to form cohesive clusters within the 3D enzyme structures. Thus, we systematically investigated the spatial distribution of residues in enzyme structures by measuring the Euclidian distances to the nearest catalytic residue for both catalytic and non-catalytic residues in individual protein sequences. As shown in Figure 2B, there was a clear difference in the distribution of Euclidian distances, with the catalytic residues peaking at ∼ 6 Å, and most non-catalytic residues exceeding 15 Å, supporting that catalytic residues form cohesive active enzymatic sites. We investigated whether SCREEN could reconstruct the same spatial distributions for enzyme catalytic residues. A performance assessment of SCREEN using the five test datasets (Figure 2C, Figure S7) showed that the majority of predicted catalytic residues were grouped together in the structure and that the distance values were consistent among the datasets, with median values being ∼ 6 Å. These results agree with findings presented in Figure 2B, implying that SCREEN accurately captures the spatial distribution of predicted catalytic residues.

These findings suggest that the use of structure information in SCREEN likely results in predictive performance improvements. We further investigated whether SCREEN could improve results compared with a “baseline model” that excludes structure-based inputs and replaces the GCN with a sequence-based convolutional neural network (CNN). The performance comparison of SCREEN and the baseline model using the five test datasets employed the Best-F1 score, AUPR, and AUC metrics (Figure 2D), and showed that the use of graph network led to a marked improvement in predictive performance.

To assess predictions, we selected enzymes from the EF-superfamily dataset that ranked around the 10%, 50%, and 90% percentiles based on the Best-F1 score. We plotted catalytic residues predicted by the structure-based SCREEN and the sequence-based baseline model against ground-truth catalytic residues within enzyme structures. In the top 10%, for carboxylic ester hydrolase (PDB ID: 1LE6), SCREEN accurately predicted all catalytic residues, whereas the sequence-based baseline model introduced three false positives (Figure 2E). In the median ranking, for casein kinase-1 (PDB ID: 1CSN), SCREEN successfully identified Asp-131, Lys-133, and Thr-181 as key residues, but the baseline model failed to identify Thr-181 (Figure 2F). In the 90% ranking, for dihydropteroate synthase (PDB ID: 1AD1), SCREEN identified Arg-239 and gave one false positive, whereas the baseline model misidentified two non-essential residues (Figure 2G). These findings indicate that SCREEN has a superior performance compared with the sequence-based baseline model. This supports our design and, in particular, the use of the graph network and enzyme structure as a key input. We also investigated various graph convolution types, including the extensively employed Graph Convolutional Layer, Graph Attention (GAT), and Graph Isomorphism Network (GIN), but none of the models outperformed another using the same test datasets and metrics (Figure S8).

### SCREEN accurately predicts catalytic residues using structure models

**Figure 3:**
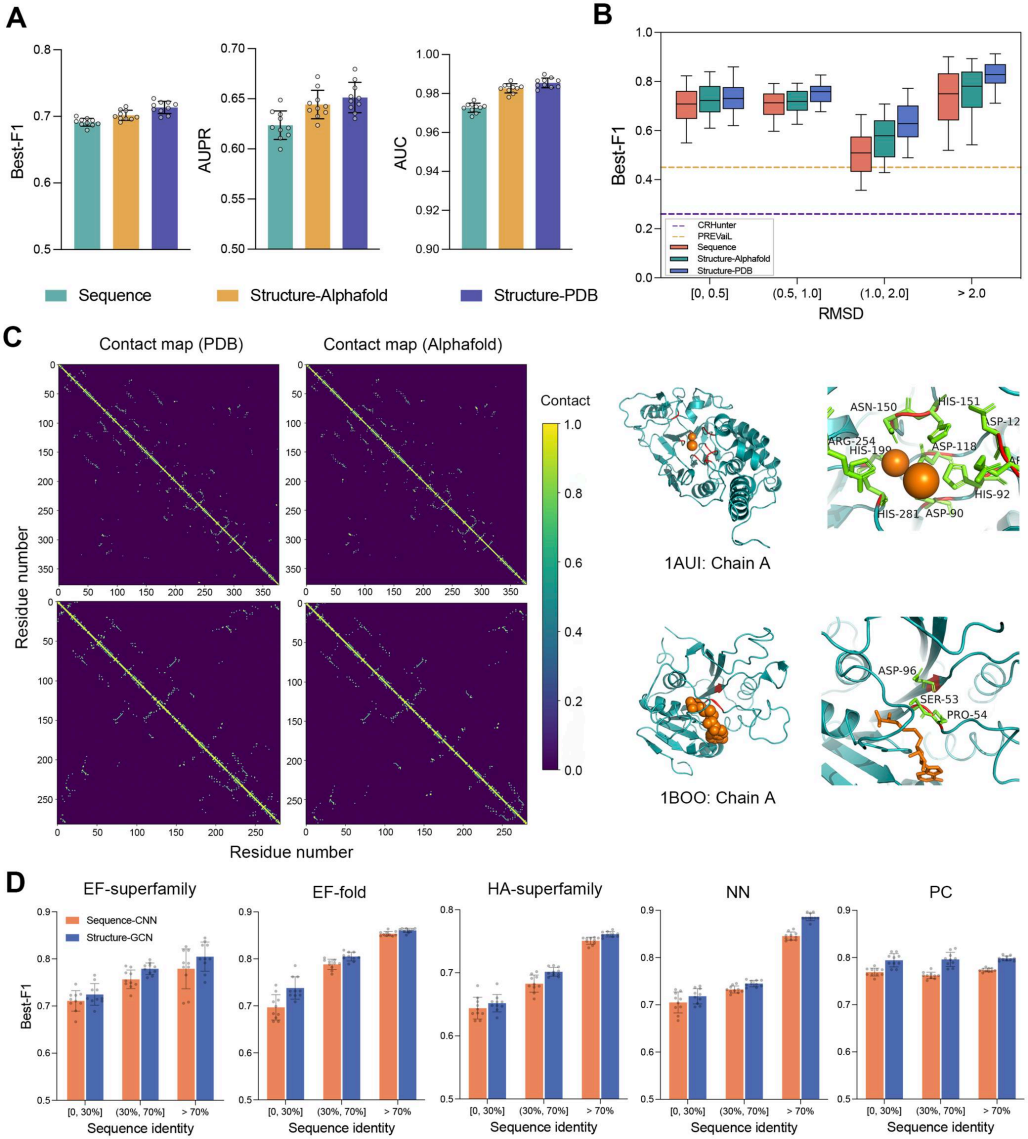
Predictive performance of SCREEN using putative enzyme structures and low-similarity test proteins **A**. Performance comparison of SCREEN using the native enzyme structures (Structure-PDB) and the AlphaFold-predicted enzyme structures (Structure-AlphaFold) with the sequence-based baseline predictor (CNN encoder). Error bars represent standard deviation of the mean based on 10 independent runs. **B**. Performance comparison of SCREEN using the native enzyme structures and the AlphaFold-predicted enzyme structures with the sequence-based baseline predictor in the context of the quality of the AlphaFold-predicted structures. The dashed horizontal lines represent the Best-F1 scores generated by CRHunter (blue line) and PREvaIL (yellow line). **C**. Examples of contact maps by ground-truth of native enzyme structures (PDB) and AlphaFold-predicted enzyme structures (left), and corresponding catalytic residues identified by SCREEN for enzymes 1AUI-A and 1BOO-A (right). **D**. Performance comparison of SCREEN with the sequence-based baseline predictor (CNN encoder) on enzymes that share varying levels of sequence identify with the training proteins on the five test datasets.

Although we showed that SCREEN’s predictive performance benefits from the use of enzyme structure data, this information is often missing for many proteins/enzymes. Recent advances in protein structure prediction, like the AlphaFold algorithm [41–43], make it possible to accurately predict protein structure from sequences and to use such structure models as the input to SCREEN. We evaluated whether the use of predicted structures rather than experimentally determined structures would alter SCREEN’s predictive performance (Table S2). We generated $C_{\alpha }$-$C_{\alpha }\;$contact maps for enzymes based on the experimental protein structures sourced from PDB, as well as the putative structures from AlphaFold. We tested SCREEN’s performance by using each of these two sets of contact maps, comparing it against a sequence-based baseline model devoid of structural information. **Figure 3**A shows that SCREEN consistently benefits from utilizing putative enzyme structures (Best-F1 = 0.702, AUPR = 0.644, and AUC = 0.985) compared with sequence data alone (Best-F1 = 0.691, AUPR = 0.624, and AUC = 0.973).

To further assess SCREEN’s denoising power on predicted structure error, we evaluated the model performance employing AlphaFold-predicted structures with varying quality. Specifically, we quantified the quality of predicted structures employing root-mean-square deviation (RMSD) metric as compared with experimental solved structures (RMSD = 0). Figure 3B reveals that SCREEN’s performance was better when employing AlphaFold-derived structures than using sequence data alone. SCREEN also outperformed currently-employed tools CRHunter [20] and PREvaIL [22] across the entire RMSD range, achieving the Best-F1 score > 0.6 using AlphaFold-predicted structures, contrasting average Best-F1 scores of 0.45 and 0.26 for CRHunter and PREvaIL, respectively.

SCREEN performed relatively well using predicted structures (Figure 3A and B), irrespective of the quality of predictions via AlphaFold. Here, we used two examples to illustrate SCREEN’s ability to accurately identify catalytic residues even in relatively low-quality predicted structures (Figure 3C). For the human calcineurin heterodimer (PDB ID: 1AUI, Chain A) [44], where the AlphaFold-predicted structure had an RMSD score of up to 6.76 Å, SCREEN successfully identified all 10 catalytic residues. Similarly, SCREEN accurately identified catalytic residues (Ser-53, Pro-54, and Asp-96) in the PVUII DNA methyltransferase (PDB ID: 1BOO, Chain A) [45], for which the structure predicted had an RMSD score of 4.75 Å. Taken together, these results suggest that SCREEN can accurately predict catalytic residues from AlphaFold-predicted enzyme structures, which might be attributed to the robustness of the input features and how they are represented in the graph network model.

### SCREEN predicts catalytic residues in “previously-unseen” enzymes

We investigated SCREEN’s ability to generate accurate predictions for “previously-unseen” enzymes. To this end, we categorized enzymes in the five test datasets into three distinct groups based on their sequence identities, namely ≤ 30% (low), 30%–70% (moderate), and > 70% (high), to enzymes in the training dataset. Figure 3D shows SCREEN’s Best-F1 scores across three sequence identity ranges, *i.e.*, ≤ 30% (low), 30%–70% (moderate), and > 70% (high), for each of the five test datasets. We showed that SCREEN consistently outperformed the sequence-based baseline model for each of the five datasets and the three identity ranges. Importantly, SCREEN’s predictions were accurate also for enzymes with limited sequence identities to those in the training datasets, achieving the Best-F1 scores of 0.725, 0.738, 0.652, 0.718, and 0.794, respectively. These predictions were significantly better than those achieved using the existing tools, such as CRHunter (Best-F1 scores of 0.328, 0.315, 0.450, 0.365, and 0.140, respectively) and PREvaIL (Best-F1 scores of 0.264, 0.261, 0.263, 0.264, and 0.263, respectively). We also used CATH [46] assignments to evaluate the model’s robustness on enzymes sharing no homologous superfamilies with those in the training dataset, as shown in Table S3 and Figure S9.

### Model training for enzyme classes improves the prediction of catalytic residues

**Figure 4:**
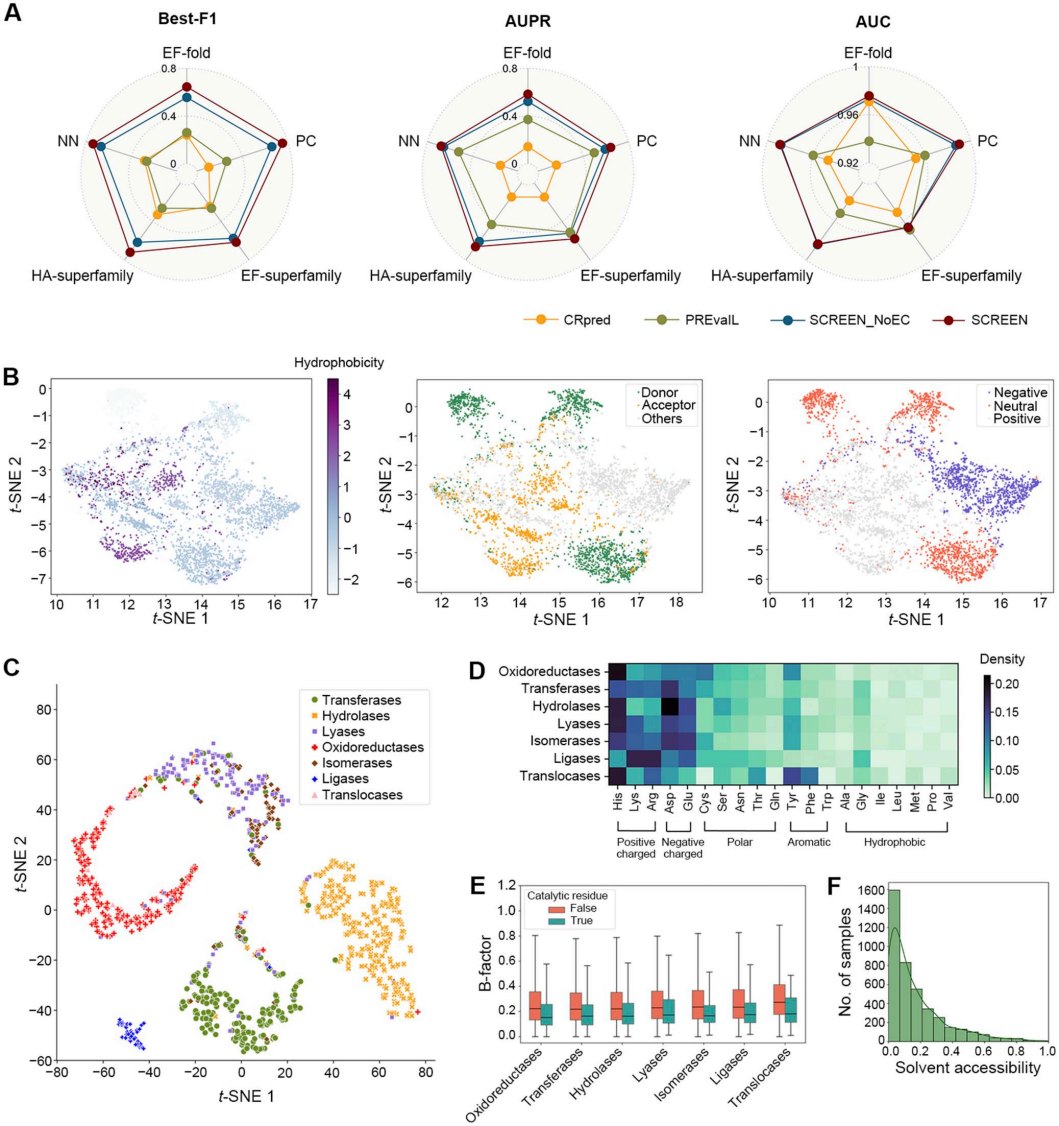
Analysis of putative catalytic residues generated by SCREEN: functional impact, latent feature spaces, and structural characteristics **A**. Performance comparison of SCREEN with its variant SCREEN_NoEC, as well as CRpred and PREvaIL. SCREEN_NoEC does not incorporate enzyme function through contrastive learning on the five test datasets. **B**. *t*-SNE-based visualization of latent feature spaces in the SCREEN model for residue characteristics, including hydrophobicity (left), hydrogen bond types (medium), and charges (right). “H-bond acceptor” denotes residues exclusively serving as H-bond acceptors without containing H-bond donor atoms. **C**. *t*-SNE-based visualization of latent feature spaces in the SCREEN model for different color-coded enzyme classes. **D**.–**F**. Analysis of structural and biophysical characteristics, including biophysical properties of amino acids (D), B-factor (E), and solvent accessibility (F) for the putative catalytic residues predicted by SCREEN. *t*-SNE, *t*-distributed stochastic neighbor embedding.

We investigated whether the training of the graph network model with distinct enzyme function implications would improve predictive performance, considering that the small subset of catalytic residues contributes to the intricate functions of enzymes. We used enzyme class information (first-level EC numbers) to refine enzyme representation through a contrastive learning framework during the training process. This led to a separation of latent feature spaces in SCREEN’s deep network model for different types of enzymes. We displayed these latent feature spaces among different enzyme classes employing *t*-distributed stochastic neighbor embedding (*t*-SNE) [47]. We showed that the predictive performance (metrics: F1, AUPR, and AUC) of SCREEN was enhanced compared to when enzyme function was not incorporated for each of the data sets (EF-fold, HA-superfamily, EF-superfamily, NN, and PC) (**Figure 4**A, Figure S10) and that SCREEN was able to group enzymes with similar functions together and separate enzymes with distinct functions (Figure 4C). Taken together, these findings indicate that the enzyme function incorporation through contrastive learning during the training process improves predictive performance as SCREEN can differentiate catalytic from non-catalytic residues for different distinct types of enzymes (Figures S11 and S12).

### SCREEN can capture selected features of catalytic residues

We analyzed catalytic residues predicted by SCREEN, in order to investigate whether they possess structural and biophysical characteristics expected for enzymes. To better understand the relevance of the features learned by SCREEN, we initially displayed the general chemical properties (including hydrophobicity, charge, and hydrogen bonds), along with low-dimensional projections of residue-level representations (Figure 4B). We observed that charged amino acids dominated in the catalytic residues predicted (Figure 4D), consistent with previous findings showing that electrostatic filtering has a marked effect on enzyme substrate selection [48]. Moreover, catalytic residues were inferred to be more rigid (structurally) than non-catalytic residues (based on low *vs.* high B-factor values; Figure 4E), which accords with a previous study of native catalytic residues [34]. Fewer hydrophobic residues were associated with catalytic residues (Figure 4D), which is consistent with limited solvent accessibility (Figure 4F) and suggests substrate avoidance in substrate–enzyme interactions [49]. Collectively, these results show that SCREEN can capture key features that typify native catalytic residues in distinct classes of enzymes.

### Linking catalytic residues to enzyme function and structure

**Figure 5:**
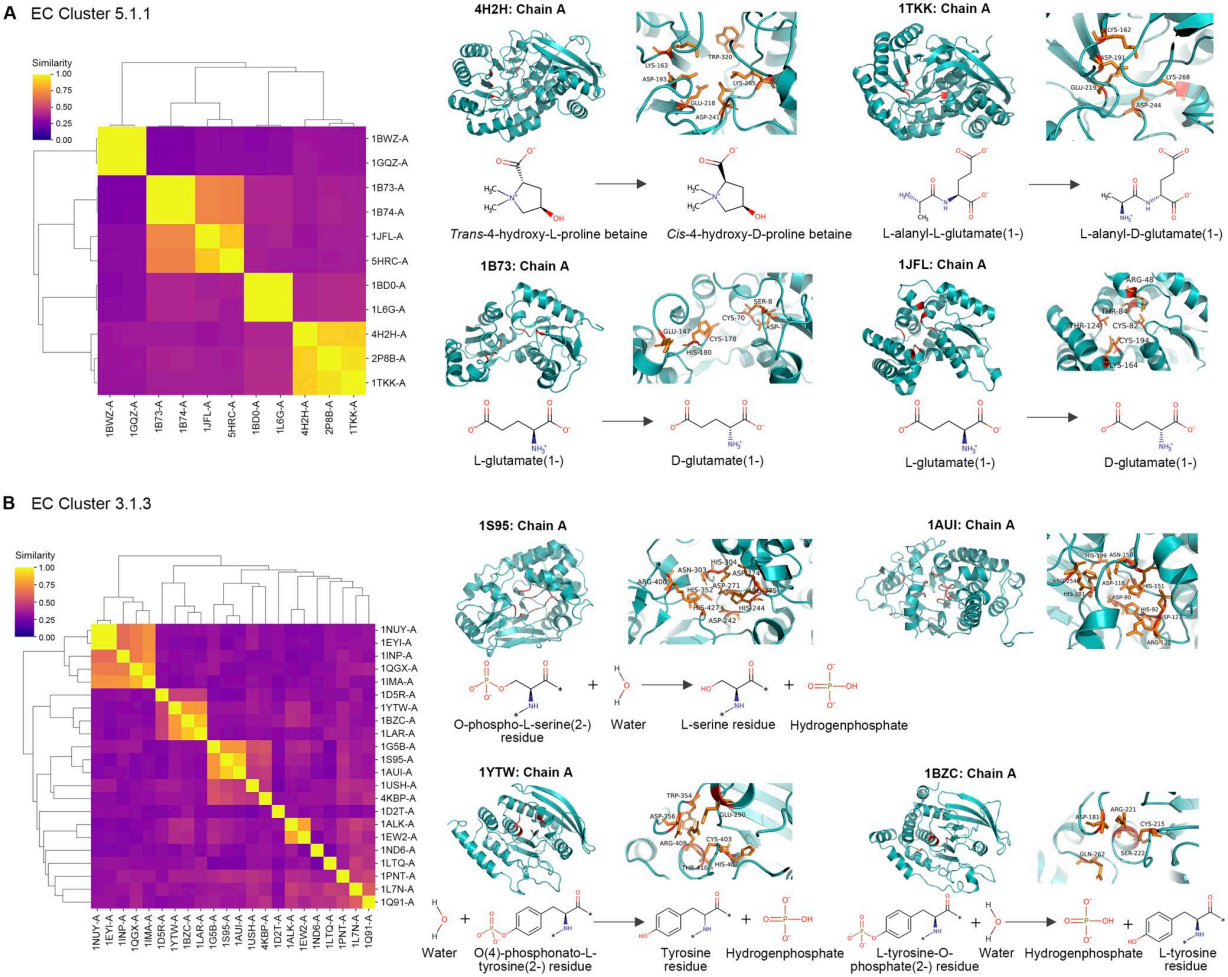
Diversity of enzyme structures and catalytic residues with the same catalytic mechanism Enzymes sharing the same catalytic mechanism were examined: isomerases acting on amino acids and derivatives with EC number 5.1.1 (**A**) and phosphoric monoester hydrolases with EC number 3.1.3 (**B**). Left: heatmaps showing the structural similarities with the TM-score as a measure. Higher values (more yellow) represent more similar structures. Right: enzyme structures with SCREEN-predicted catalytic residues mapped (top) as well as enzyme reactions (bottom).

Based on these catalytic residues, we further analyzed the sequence-structure-function relationship of enzymes to gain deeper insights into enzymes’ catalytic mechanisms. We categorized enzymes according to their catalytic functions defined by third-level EC numbers and then by (complete) fourth-level EC numbers (which link to substrates). For enzyme clusters sharing the same catalytic function/mechanism, we assessed structural similarity by their TM-scores among cluster members [50], selected enzymes from individual clusters, and mapped the catalytic residue predictions to respective 3D structures (**Figure 5**).

The results indicated that same catalytic motif may represent distinct enzymes that serve different functions linked to diverse ligands or substrates. Figure 5A shows that both 4-hydroxyproline betaine 2-epimerase (PDB ID: 4H2H, Chain A) and L-alanyl-D/L-glutamate epimerase (PDB ID: 1TKK, Chain A) belong to the same superfamily and share a common catalytic motif (KDEDK) [51], but they differ in their substrate specificity: 4-hydroxyproline betaine 2-epimerase facilitates the 2-epimerization of *trans*-4-hydroxy-L-proline betaine (tHyp-B) to *cis*-4-hydroxy-D-proline betaine (cHyp-B), whereas L-alanyl-D/L-glutamate epimerase catalyzes the reversible epimerization of L-alanyl-D-glutamate to L-alanyl-L-glutamate.

Enzymes in a particular family can have similar structures and functions, despite undergoing sequence divergence through evolution. A compelling illustration emerges when we studied two related enzymes, the glutamate racemase (PDB ID: 1B73, Chain A) [52] and aspartate racemase (PDB ID: 1JFL, Chain A) [53] (Figure 5A). Despite enabling similar reactions via the same mechanism, their catalytic residues are significantly different but have analogous tertiary structures. Another example relates to protein-tyrosine-phosphatase non-receptor class (PDB ID: 1YTW, Chain A) [54] and protein-tyrosine-phosphatase non-receptor type 1 (PDB ID: 1BZC, Chain A) [55] (Figure 5B). Here, although both enzymes are tyrosine phosphatases and catalyze the same reaction to remove phosphoryl groups from tyrosine residues in proteins, their respective catalytic residues are distinctly different.

The shared structural arrangements of catalytic residues can be associated with functional similarity. Figure 5B shows that phosphatase 5 (PDB ID: 1S95, Chain A) and phosphatase 2B (PDB ID: 1AUI, Chain A) exhibit significant structural similarity and share catalytic residues (motif: DHDDRNHHRH) pertaining to serine/threonine phosphatase function(s), characterized by executing a “nucleophilic assault” on the phosphorus atom within a phosphorylated serine or threonine residue [44].

Although enzymes catalyzing the same reactions often exhibit marked sequence and/or structural similarity, exceptions exist where structurally dissimilar enzymes enable similar reactions via the same mechanism. This is expected since enzymes facilitate numerous reactions using a finite set of building blocks in their residues, resulting in multiple enzymes inevitably sharing components of their catalytic mechanisms. Here, we showed that non-homologous proteins, protein phosphatase 5 (PDB ID: 1S95, Chain A) and dual-specificity phosphatase (PDB ID: 1D5R, Chain A) employ distinct structural motifs to execute the same reaction that dephosphorylates a phosphoprotein substrate (Figure 5B, Figure S13).

### Associating mutations with the SCREEN-predicted catalytic pockets

Here, we studied the patterns of mutations in the context of their proximity to the catalytic pockets predicted by SCREEN. We collected the MAVE data, probing mutation effect(s) on the functions of four different enzymes, namely PTEN tumor suppressor (PDB ID: 1D5R, Chain A) [56], human cytochrome P450 CYP2C9 (PDB ID: 1OG5, Chain A) [57], NUDT15 (PDB ID: 5LPG, Chain A) [58], and *Escherichia coli* TEM1 beta-lactamase (PDB ID: 1BTL, Chain A) [59], encompassing a total of 15,665 variants across 1343 residues.

To function effectively, enzymes must be present at sufficiently high levels and have suitable catalytic residues in the active sites [60]; however, mutations can affect both of these aspects, potentially resulting in impaired enzymatic function. We used the MAVE data [61] for two residue classes: (1) WTL residues that exhibit high functional tolerance to mutations, whose most missense mutations do not adversely impact enzyme function; and (2) FL residues that are prone to mutations that either decrease abundance (*e.g.*, unstable structures) and/or impair function, leading to diminished enzyme activity (Figure S14).

**Figure 6:**
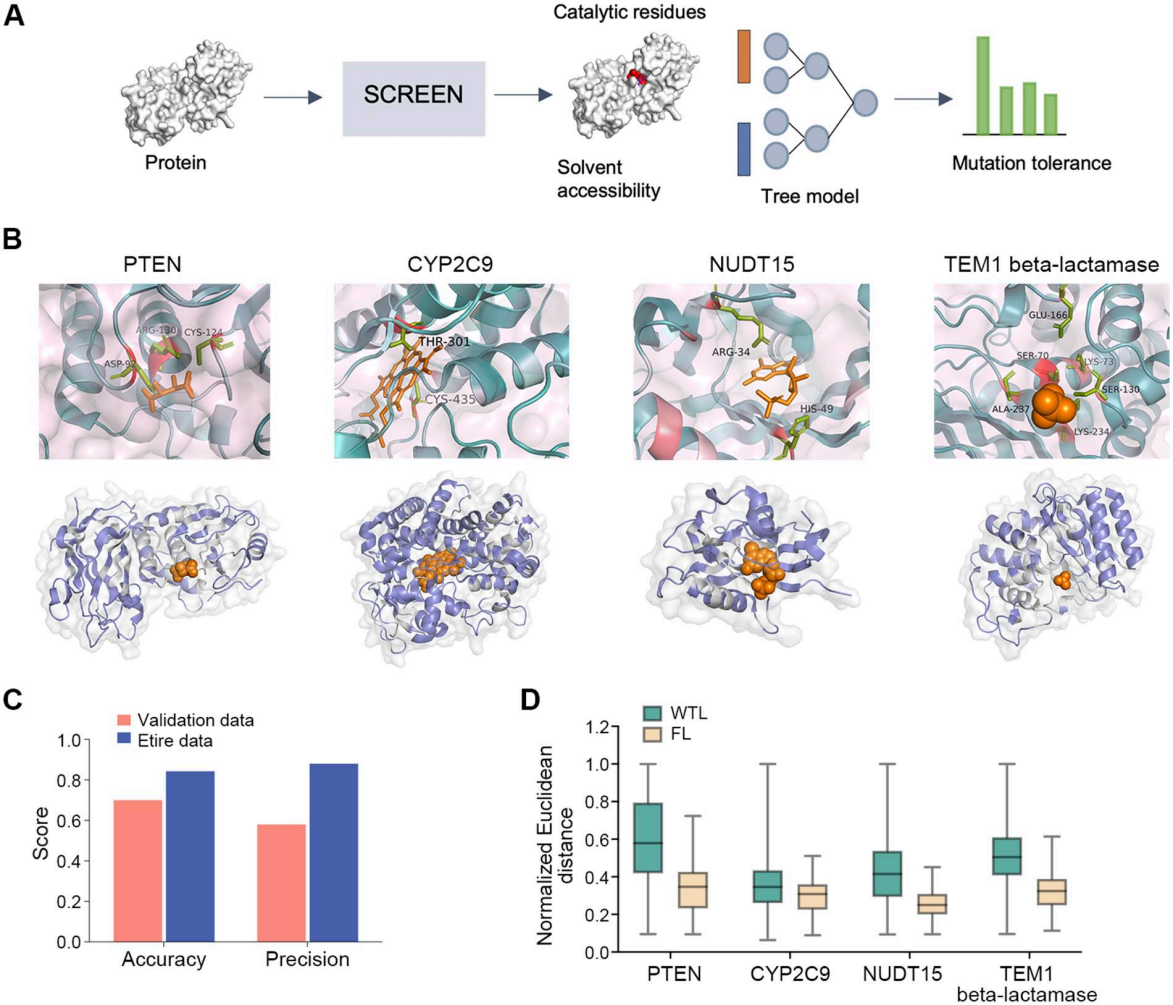
Assessing the tolerance of catalytic residues to mutations **A**. Architecture of the tree-structured model for characterizing mutated residues based on catalytic residue predictions by SCREEN. **B**. Top: SCREEN-identified catalytic residues of four different enzymes: PTEN tumor suppressor (PDB ID: 1D5R, Chain A), human cytochrome P450 CYP2C9 (PDB ID: 1OG5, Chain A), NUDT15 (PDB ID: 5LPG, Chain A), and *Escherichia coli* TEM1 beta-lactamase (PDB ID: 1BTL, Chain A). Bottom: residues within enzyme structures are colored according to their predicted mutation classes. Purple represents WTL residues, gray denotes FL residues, and orange corresponds to the substrate. **C**. Quality of distance-based classification of residues within different mutation classes, measured by accuracy and precision on both the validation and entire datasets. **D**. Distribution of the Euclidean distances for residues of different mutation classes. WTL, wild type-like; FL, functional loss.

We systematically analyzed key characteristics of mutations in the context of the predicted catalytic residues. Specifically, we applied an additional tree-structured model to SCREEN (**Figure 6**A). The Euclidean distances to the closest catalytic residue predicted by SCREEN combined with solvent accessibility, which, as expected, were inferred to vary according to residue type (WTL or FL), allowing to differentiate among different mutation groups. Figure 6B illustrates the catalytic residues predicted by SCREEN along with residue mutation type predictions. We performed five-fold cross-validation on all MAVE data; our results revealed an average accuracy of 0.70 and 0.84, and precision of 0.58 and 0.88 on the validation and entire datasets, respectively (Figure 6C). We found distinct spatial distribution patterns for the WTL and FL residues based on their Euclidean distances from the putative catalytic residues (Figure 6D). Our result aligns well with experimental data showing that FL residues are relatively close to the catalytic site, while WTL residues are distributed throughout the structure (Figure S15). This result indicates that SCREEN can be useful to establish the impact of mutations on enzyme structure and function and provides a tool to guide the identification of disease-associated mutations in enzymes.

## Discussion

Enzymes can catalyze a broad set of chemical reactions using a limited set of catalytic residues [62]. Identifying these residues allows us to understand how existing enzymes function at the molecular level and to design new ones. In this work, we hypothesize that the structural organization of catalytic residues in spatial space, along with their generally high evolutionary conservation, collectively contributes to catalytic residue identification. To this end, we conceptualized, designed, and assessed SCREEN, a structure-based graph network that uses functional priors through contrastive learning and combines structure-, sequence-, and evolutionary profile-based representations to accurately predict catalytic residues in enzymes.

Comparative empirical assessments using five commonly-utilized test datasets and seven currently-available (published) predictors revealed that SCREEN (1) accurately predicts catalytic residues in known and computationally-modeled enzymes; (2) outperforms current tools; and (3) generalizes well to enzymes that have limited similarity to enzymes used to train the model, suggesting that SCREEN is applicable to currently-unknown enzymes. Incorporating enzyme function as a prior could improve the prediction of catalytic residues by enhancing the consistency of enzyme’s latent representations based on their functions. However, we did not explore enzyme function prediction in depth, *e.g.*, addressing questions such as can we also utilize this model to solve enzyme function classification tasks? A more comprehensive understanding of the spatial distribution across diverse enzyme functions and the sequence-structure-function relationship could be achieved by analyzing a larger sample of enzymes that thoroughly covers the EC space. Additionally, our analysis is limited by treating enzymes as independent units. Enzymatic reactions involve multiple residues, substrates, and cofactors interacting across various chemical steps [13]; as such, an integrated analysis would be necessary for a more comprehensive understanding of the catalytic chemical activity. Further, future research should focus on determining the level of confidence that can be assigned to model predictions of catalytic residues, as well as exploring the techniques that can effectively assess this confidence.

We demonstrate that SCREEN could infer key structural and biophysical features, including amino acid charge, solvent accessibility, and structural rigidity, of predicted and known catalytic residues. Further, we undertook sequence-structure-function analyses to link catalytic residues to enzyme structure and function. Our analyses revealed that while enzymes that catalyze identical reactions often display significant sequence and/or structural similarity, exceptions arise wherein dissimilar sequences and/or structures can catalyze reactions via the same mechanism. In addition, using multiplexed enzyme mutation data, we showed that SCREEN could infer the tolerance of individual catalytic residues to mutations and, thus, predict which mutations in catalytic residues likely lead to the functional loss of an enzyme. Taken together, SCREEN should provide a useful tool for the reliable prediction of catalytic residues to support studies of known and unknown enzyme groups/classes as well as enable *in silico* investigations of diseases linked to mutations.

## Conclusion

SCREEN is an efficient and robust method for high-throughput prediction of catalytic residues by integrating enzyme functional and structural information. We demonstrate SCREEN’s effectiveness and robustness across various widely used datasets, illustrating that the predicted putative catalytic residues closely align with the key structural and biophysical characteristics of native catalytic residues. Furthermore, we performed sequence-structure-function analyses to establish connections between catalytic residues and enzyme structure and function. This highlights SCREEN’s potential for reliably predicting catalytic residues in both known and unknown enzyme groups/classes, thereby supporting studies of the molecular mechanisms underlying enzyme functions.

## Code availability

All source code is available at GitHub (https://github.com/BioColLab/SCREEN). The source code has also been submitted to BioCode at the National Genomics Data Center, Beijing Institute of Genomics, Chinese Academy of Sciences / China National Center for Bioinformation (BioCode: BT007580), which is publicly accessible at https://ngdc.cncb.ac.cn/biocode/tool/7580.

## Supplementary Material

qzae094_Supplementary_Data

## Data Availability

All source data needed to evaluate the conclusions in this study can be found at https://huggingface.co/datasets/Biocollab/SCREEN/tree/main.

## CRediT author statement

**Tong Pan:** Conceptualization, Methodology, Investigation, Writing – original draft. **Yue Bi:** Visualization. **Xiaoyu Wang:** Visualization. **Ying Zhang:** Visualization. **Geoffrey I. Webb:** Methodology, Supervision. **Robin B. Gasser:** Investigation, Supervision, Writing – review & editing. **Lukasz Kurgan:** Methodology, Investigation, Supervision, Writing – review & editing. **Jiangning Song:** Conceptualization, Methodology, Investigation, Supervision, Writing – review & editing. All authors have read and approved the final manuscript.
