# Supplementary material for: SCREEN: A Graph-based Contrastive Learning Tool to Infer Catalytic Residues and Assess Enzyme Mutations
Source: Genomics Proteomics Bioinformatics. 2024 Dec 26;22(6):qzae094. doi: 10.1093/gpbjnl/qzae094 (PMC11961199; doi:10.1093/gpbjnl/qzae094)
Supplement: qzae094_Supplementary_Data [file qzae094_supplementary_data.zip › Table S3 120824.docx]

**Table S3 Comparison with enzymes from novel superfamilies across five test datasets**

| **Metrics** | **Data** | **EF**  **Superfamily**  **dataset** | **EF fold**  **dataset** | **HA superfamily**  **dataset** | **NN**  **dataset** | **PC**  **dataset** |
| --- | --- | --- | --- | --- | --- | --- |
| AUPR | SCREEN  (all data) | 0.580 | 0.584 | 0.661 | 0.658 | 0.634 |
|  | SCREEN (novel superfamily) | 0.525 | 0.511 | 0.362 | 0.493 | 0.578 |
| AUC | SCREEN  (all data) | 0.968 | 0.976 | 0.983 | 0.989 | 0.990 |
|  | SCREEN (novel superfamily) | 0.960 | 0.969 | 0.943 | 0.969 | 0.976 |
| F1 | SCREEN  (all data) | 0.615 | 0.645 | 0.720 | 0.738 | 0.741 |
|  | SCREEN (novel superfamily) | 0.571 | 0.610 | 0.450 | 0.565 | 0.639 |
