# Supplementary material for: SCREEN: A Graph-based Contrastive Learning Tool to Infer Catalytic Residues and Assess Enzyme Mutations
Source: Genomics Proteomics Bioinformatics. 2024 Dec 26;22(6):qzae094. doi: 10.1093/gpbjnl/qzae094 (PMC11961199; doi:10.1093/gpbjnl/qzae094)
Supplement: qzae094_Supplementary_Data [file qzae094_supplementary_data.zip › Table S2 120824.docx]

**Table S2 Comparison with ESM-predicted structure**

| **Metrics** | **Methods** | **EF**  **Superfamily**  **dataset** | **EF fold**  **dataset** | **HA superfamily**  **dataset** | **NN**  **dataset** | **PC**  **dataset** |
| --- | --- | --- | --- | --- | --- | --- |
| AUPR | SCREEN (This study) | 0.580 | 0.584 | 0.661 | 0.658 | 0.634 |
|  | SCREEN (ESM-structure) | 0.546 | 0.509 | 0.609 | 0.664 | 0.667 |
| AUC | SCREEN (This study) | 0.968 | 0.976 | 0.983 | 0.989 | 0.990 |
|  | SCREEN (ESM-structure) | 0.964 | 0.972 | 0.979 | 0.989 | 0.990 |
| F1 | SCREEN (This study) | 0.615 | 0.645 | 0.720 | 0.738 | 0.741 |
|  | SCREEN (ESM-structure) | 0.584 | 0.601 | 0.670 | 0.699 | 0.719 |
