# Supplementary material for: SCREEN: A Graph-based Contrastive Learning Tool to Infer Catalytic Residues and Assess Enzyme Mutations
Source: Genomics Proteomics Bioinformatics. 2024 Dec 26;22(6):qzae094. doi: 10.1093/gpbjnl/qzae094 (PMC11961199; doi:10.1093/gpbjnl/qzae094)
Supplement: qzae094_Supplementary_Data [file qzae094_supplementary_data.zip › Figure S3.pdf]

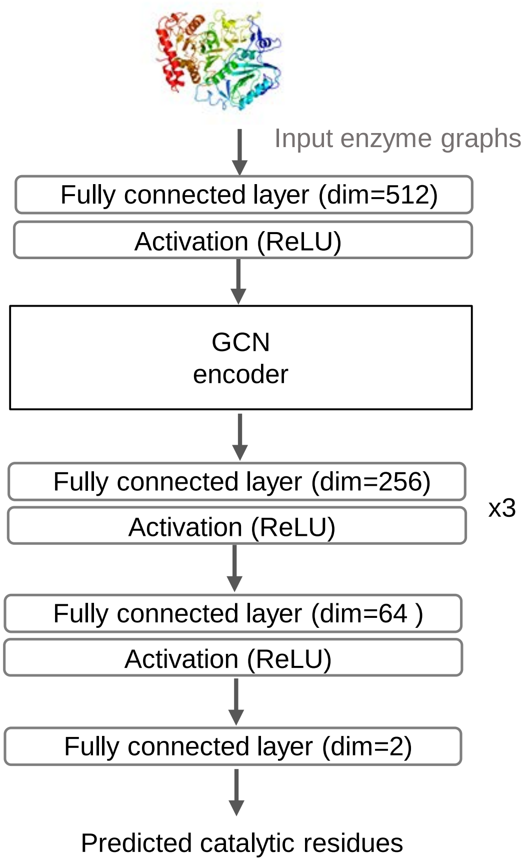
Input enzyme graphs
Fully connected layer (dim=512)
Activation (ReLU)
GCN
encoder
Fully connected layer (dim=256)
Activation (ReLU)
x3
Fully connected layer (dim=64)
Activation (ReLU)
Fully connected layer (dim=2)
Predicted catalytic residues
