# Supplementary material for: SCREEN: A Graph-based Contrastive Learning Tool to Infer Catalytic Residues and Assess Enzyme Mutations
Source: Genomics Proteomics Bioinformatics. 2024 Dec 26;22(6):qzae094. doi: 10.1093/gpbjnl/qzae094 (PMC11961199; doi:10.1093/gpbjnl/qzae094)
Supplement: qzae094_Supplementary_Data [file qzae094_supplementary_data.zip › Figure S12.pdf]

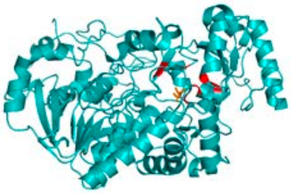

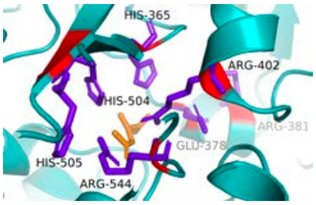

PDB 1qjd-A
Oxidoreductases

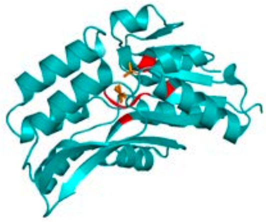

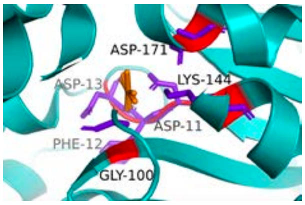

PDB 1l7n-A
Hydrolases

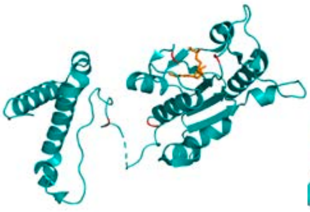

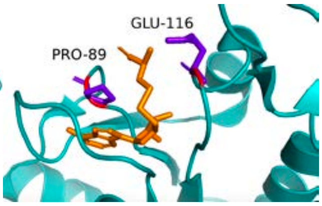

PDB 1uam-A
Transferases

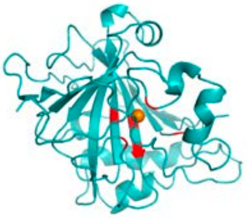

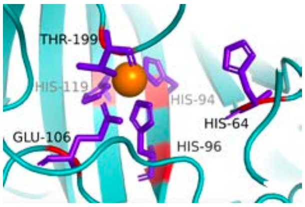

PDB 1ca2-A
Lyases

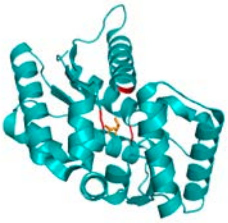

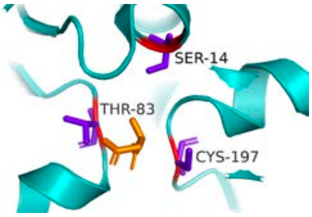

PDB 5hrc-A
Isomerases

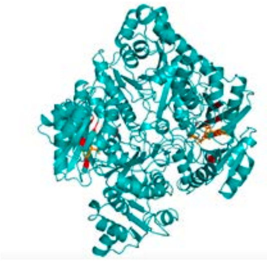

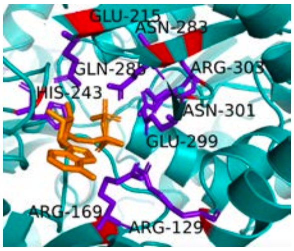

PDB 1bxr-A
Ligases

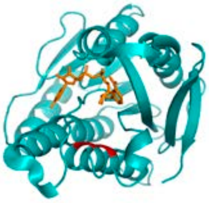

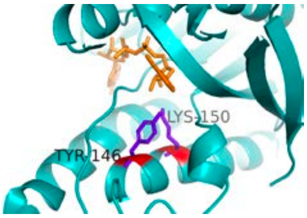

PDB 1dhr-A
Translocases
