# Supplementary material for: SCREEN: A Graph-based Contrastive Learning Tool to Infer Catalytic Residues and Assess Enzyme Mutations
Source: Genomics Proteomics Bioinformatics. 2024 Dec 26;22(6):qzae094. doi: 10.1093/gpbjnl/qzae094 (PMC11961199; doi:10.1093/gpbjnl/qzae094)
Supplement: qzae094_Supplementary_Data [file qzae094_supplementary_data.zip › Table S1 120824.docx]

**Table S1**  **Training, validation and test datasets**

| **Type** | **Dataset name** | **Enzymes** | **Catalytic residues** | **Non-catalytic residues** | **Ratio** |
| --- | --- | --- | --- | --- | --- |
| Training and internal validation | Curated M-CSA | 762 | 3344 | 272287 | \| 1:81 \| \| --- \| \|  \| |
|  | EF family | 293 | 1018 | 86013 | \| 1:84 \| \| --- \| |
| Test  (comparative evaluation against current methods) | EF fold | 189 | 605 | 48223 | \| 1:80 \| \| --- \| |
|  | EF superfamily | 236 | 716 | 62677 | \| 1:87 \| \| --- \| |
|  | HA superfamily | 282 | 933 | 97976 | \| 1:105 \| \| --- \| |
|  | NN | 163 | 599 | 56309 | \| 1:94 \| \| --- \| |
|  | PC | 81 | 289 | 24493 | 1:85 |
