# Supplementary material for: SCREEN: A Graph-based Contrastive Learning Tool to Infer Catalytic Residues and Assess Enzyme Mutations
Source: Genomics Proteomics Bioinformatics. 2024 Dec 26;22(6):qzae094. doi: 10.1093/gpbjnl/qzae094 (PMC11961199; doi:10.1093/gpbjnl/qzae094)
Supplement: qzae094_Supplementary_Data [file qzae094_supplementary_data.zip › supplementary material captions 121424.docx]

**Supplementary material**

**Figure S1 Primary EC number-based categorization of enzymes in the training, validation and the five test datasets**

Enzymes in the training, validation, and five test datasets are grouped by their primary EC number, showing the enzyme counts and proportions within each functional category.

**Figure S2 Architecture of the proposed enzyme function classification pipeline**

SCREEN features two core pipelines. The first pipeline employs a contrastive learning framework to establish an enzyme function classification module, using Enzyme Commission (EC) numbers to define enzyme classes. This module aims to leverage top-level EC functional classes as cues to implicitly learn refined enzyme embeddings.

**Figure S3 Architecture of the proposed catalytic residue prediction pipeline**

This pipeline predicts catalytic residues by representing the input enzyme as an attributed graph, with residues as nodes. The feature matrix $X$= ($X_{L},X_{G},X_{A}$) incorporates evolutionary conservation (via PSI-BLAST and HMMER), sequence embeddings (via ProtT5), and atomic-level properties, including atom types, atomic mass (excluding hydrogen), B-factor, side-chain presence, bonded hydrogen atom count, ring membership, van der Waals radius, and solvent accessibility.

**Figure S4 Comparison of the SCREEN performance trained with different hidden dimensions**

The hypermeter of hidden_dimension in SCREEN was set to 512. We compare performance of the SCREEN models with hidden_dimension = 256 (SCREEN_256) and hidden_dimension = 1024 (SCREEN_1024). The SCREEN with the default dimension (SCREEN_512) achieves better performance in terms of Best-F1 score, AUC and AUPR metrics.

**Figure S5 The importance of different input features for SCREEN Performance on five test datasets**

Feature importance was assessed by removing specific features and evaluating their impact on model performance. Considered features included evolutionary conservation (PSSM from PSI-BLAST and HMM from HMMER), residue-level features (sequence information from the ProtT5 model), and atomic-level features.

**Figure S6 SCREEN performance for each enzyme function category across the five test datasets**

**Figure S7 Performance of SCREEN trained with different contact map distance thresholds (6Å, 8 Å, and 10 Å) on the five test datasets**

Data are represented as boxplots with the center line representing the median, upper and lower edges of the boxes representing the interquartile range, and whiskers representing the data range (0.5 x interquartile range).

**Figure S8 Performance of SCREEN with different GNN layers on the five test datasets, in comparison to the sequence-only CNN**

Three commonly used GNN layers (GCN, GIN, and GAT) are considered. AUPR values across ten independent runs are presented, with error bars indicating the standard deviation of the mean.

**Figure S9 Categorization of enzymes into novel primary superfamilies using CATH across five test datasets (left), performance of SCREEN on enzymes from these novel superfamilies (right)**

**Figure S10 Performance comparison of SCREEN with its variant that does not apply contrastive learning (SCREEN_Noec) across different enzyme classes on the five test datasets**

**Figure S11 The visualization of latent feature spaces in the SCREEN model for different color-coded enzyme residues (catalytic residues in blue and non-catalytic residues in red)**

**Figure S12 Catalytic residue predictions by SCREEN mapped onto the 3D structure for enzymes with seven distinct functions**

The predicted catalytic sites are in red, while putative non-catalytic residues are in cyan.

**Figure S13 A detailed view of catalytic residues (top), with the ligand in cyan, catalytic residues in pink, and residues located within 10 Å of the catalytic residues (bottom) of PTEN, with the ligand in pink, catalytic residues in purple, and mutated residues in yellow**

**Figure S14 Protocol used to define thresholds for the multiplexed data generated by MAVEs**For each enzyme, we fit the score distributions from each MAVE (light blue) to a mixture model with three Gaussian components (black line) and use the intersection of the first and last Gaussians as the cut-off.

**Figure S15 Enzyme structures with residues colored according to their mutant class: blue corresponds to the Wild Type-like (WTL) residues, gray to the Functional Loss (FL) residues**

**Table S1 Training, validation and test datasets**

**Table S2 Comparison with ESM-predicted structure**

**Table S3 Comparison with enzymes from novel superfamilies across five test datasets**
