# Supplementary figures and images for: SCREEN: A Graph-based Contrastive Learning Tool to Infer Catalytic Residues and Assess Enzyme Mutations
Source: Genomics Proteomics Bioinformatics. 2024 Dec 26;22(6):qzae094. doi: 10.1093/gpbjnl/qzae094 (PMC11961199; doi:10.1093/gpbjnl/qzae094)

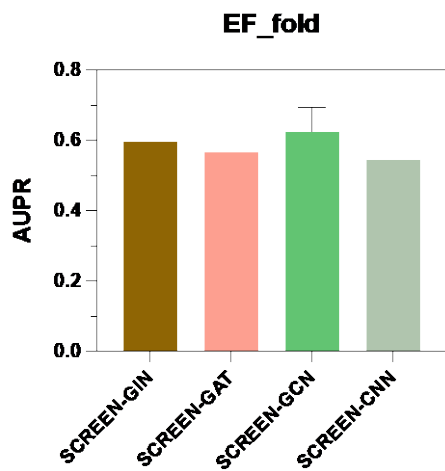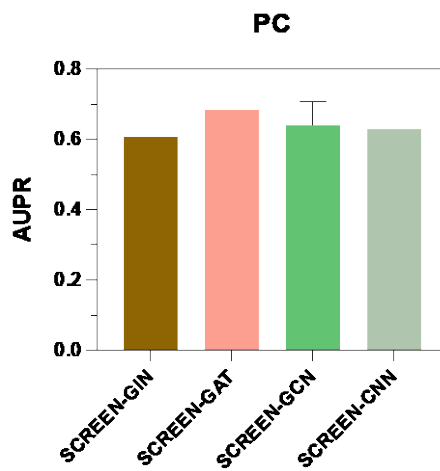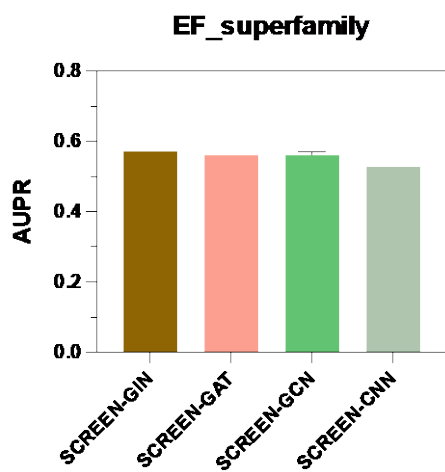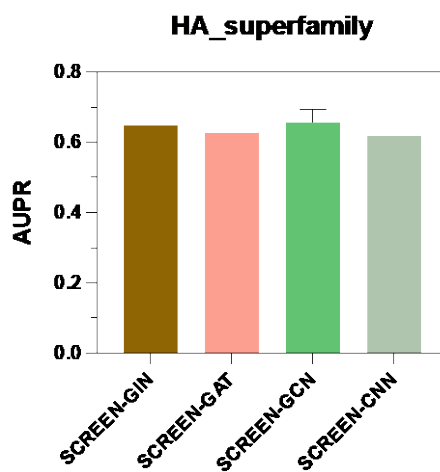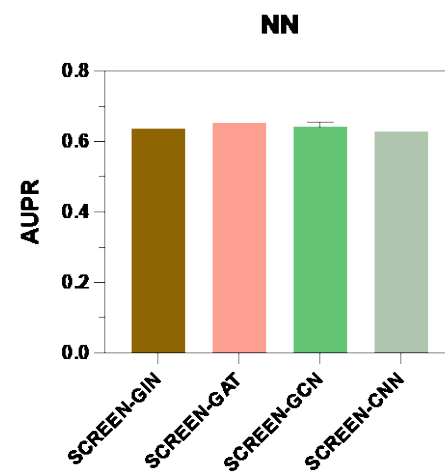

Supplement: qzae094_Supplementary_Data [file qzae094_supplementary_data.zip › Figure S8.pdf]

EF\_superfamily

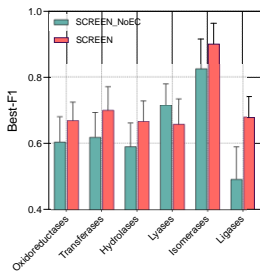

EF\_fold

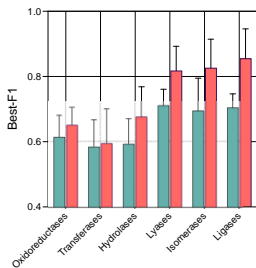

PC

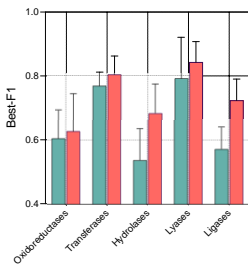

HA\_superfamily

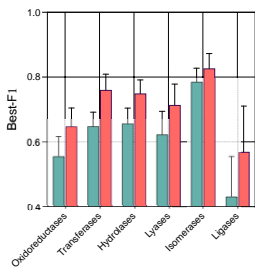

NN

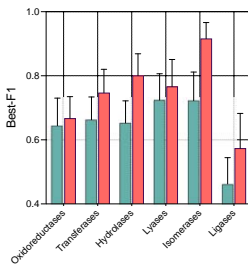

Supplement: qzae094_Supplementary_Data [file qzae094_supplementary_data.zip › Figure S10.pdf]

**NN**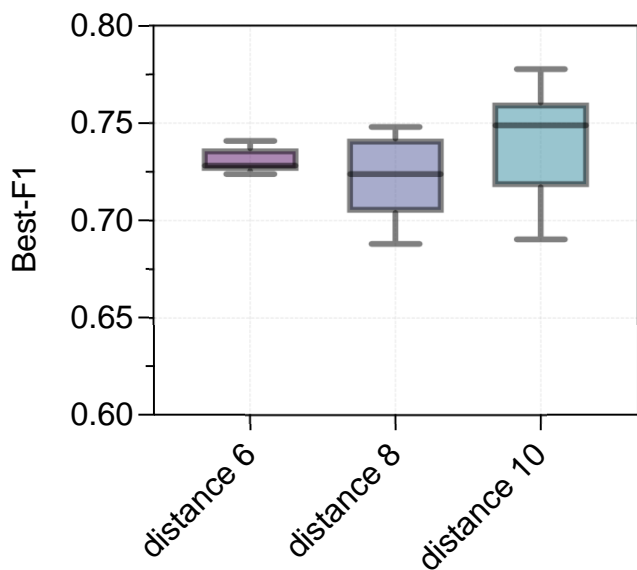**HA\_superfamily**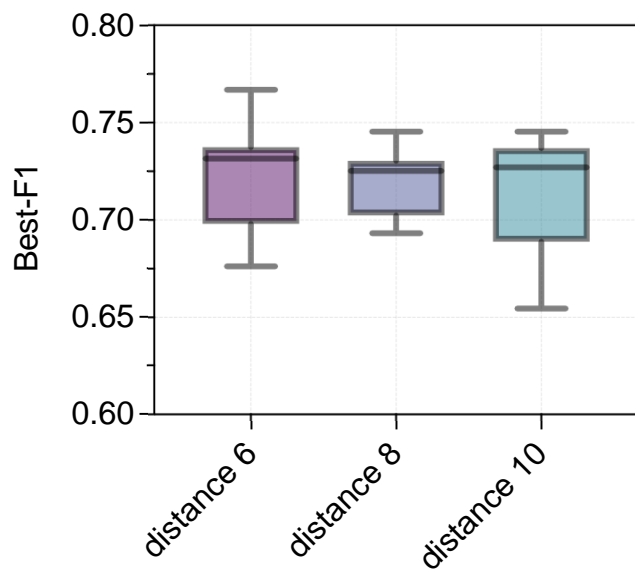**EF\_superfamily**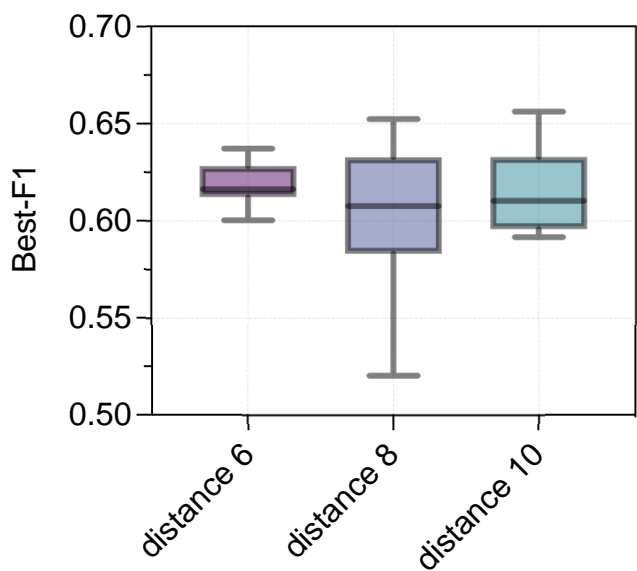**PC**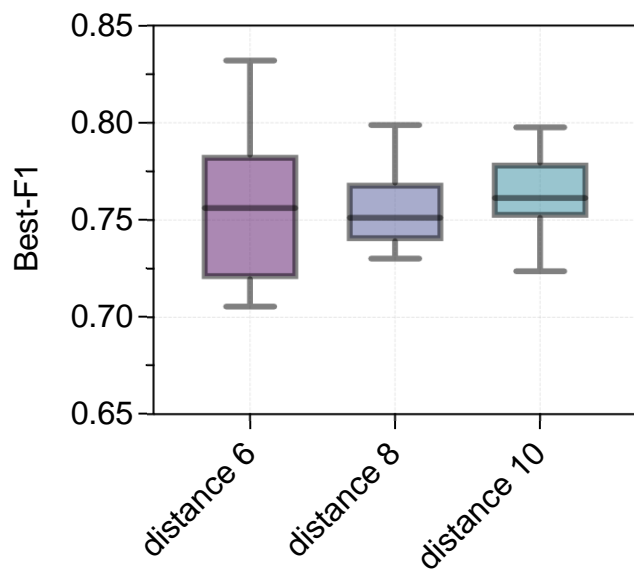**EF\_fold**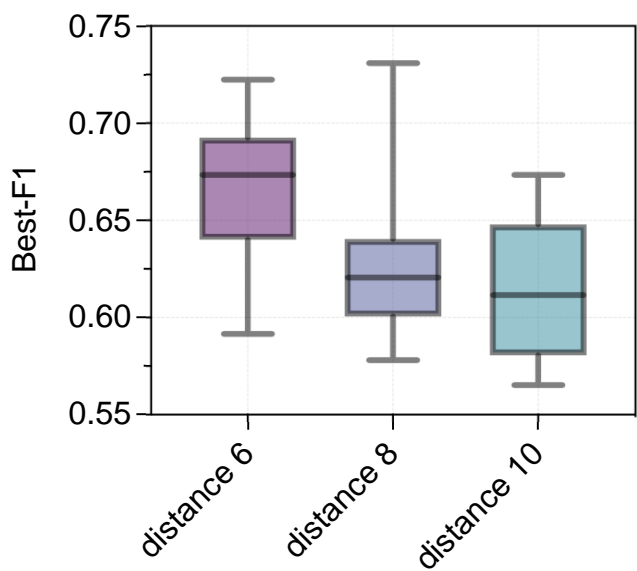

Supplement: qzae094_Supplementary_Data [file qzae094_supplementary_data.zip › Figure S7.pdf]

**Best-F1**

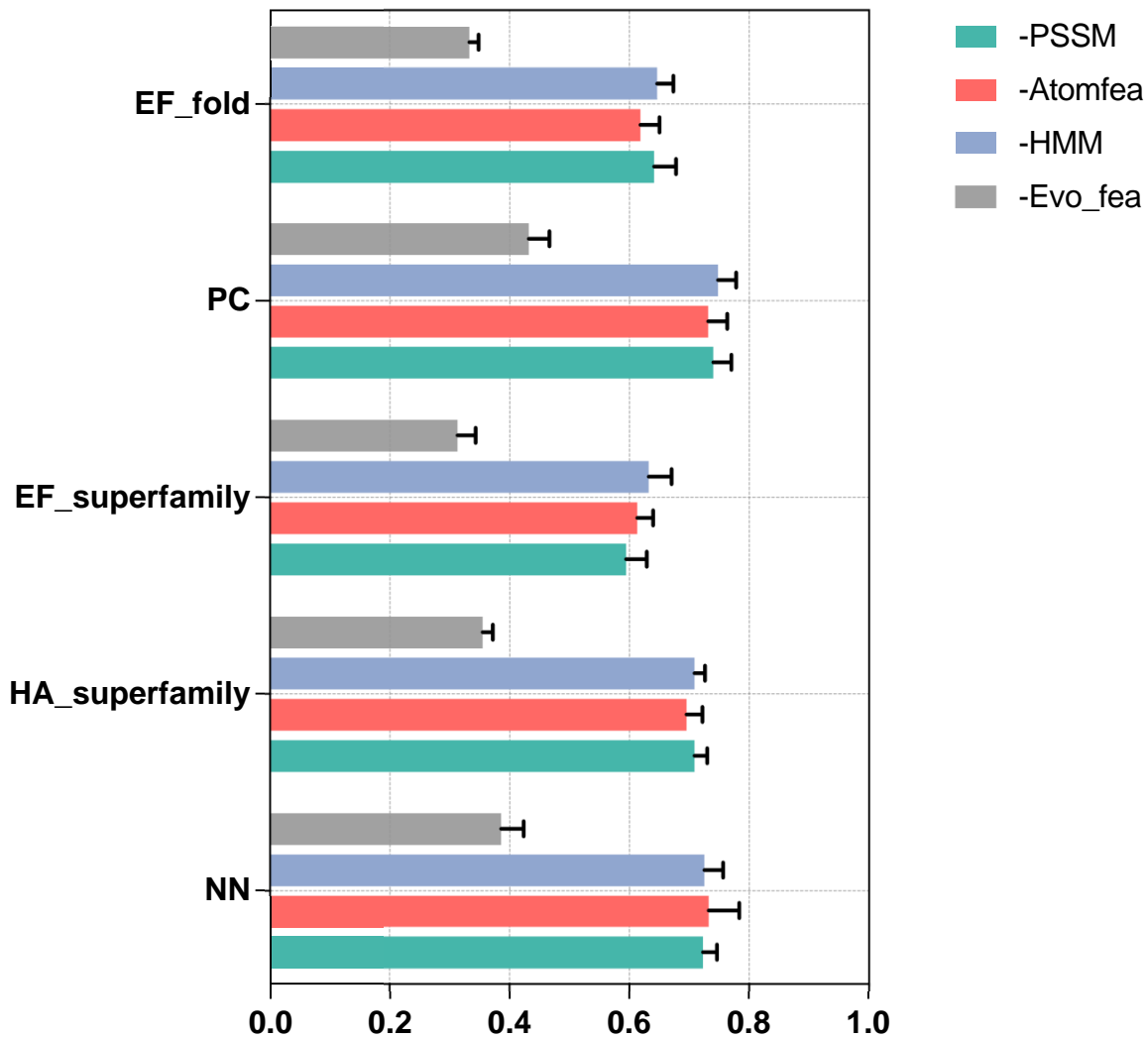

Supplement: qzae094_Supplementary_Data [file qzae094_supplementary_data.zip › Figure S5.pdf]

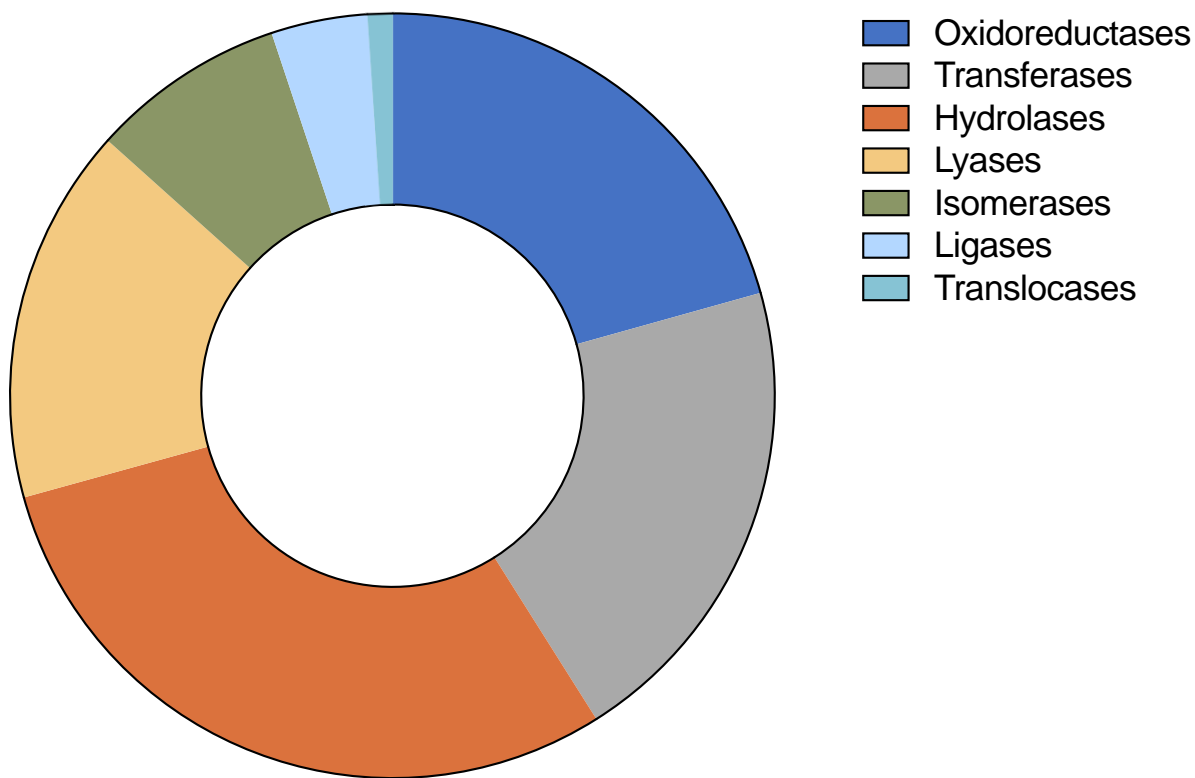

Total=1055

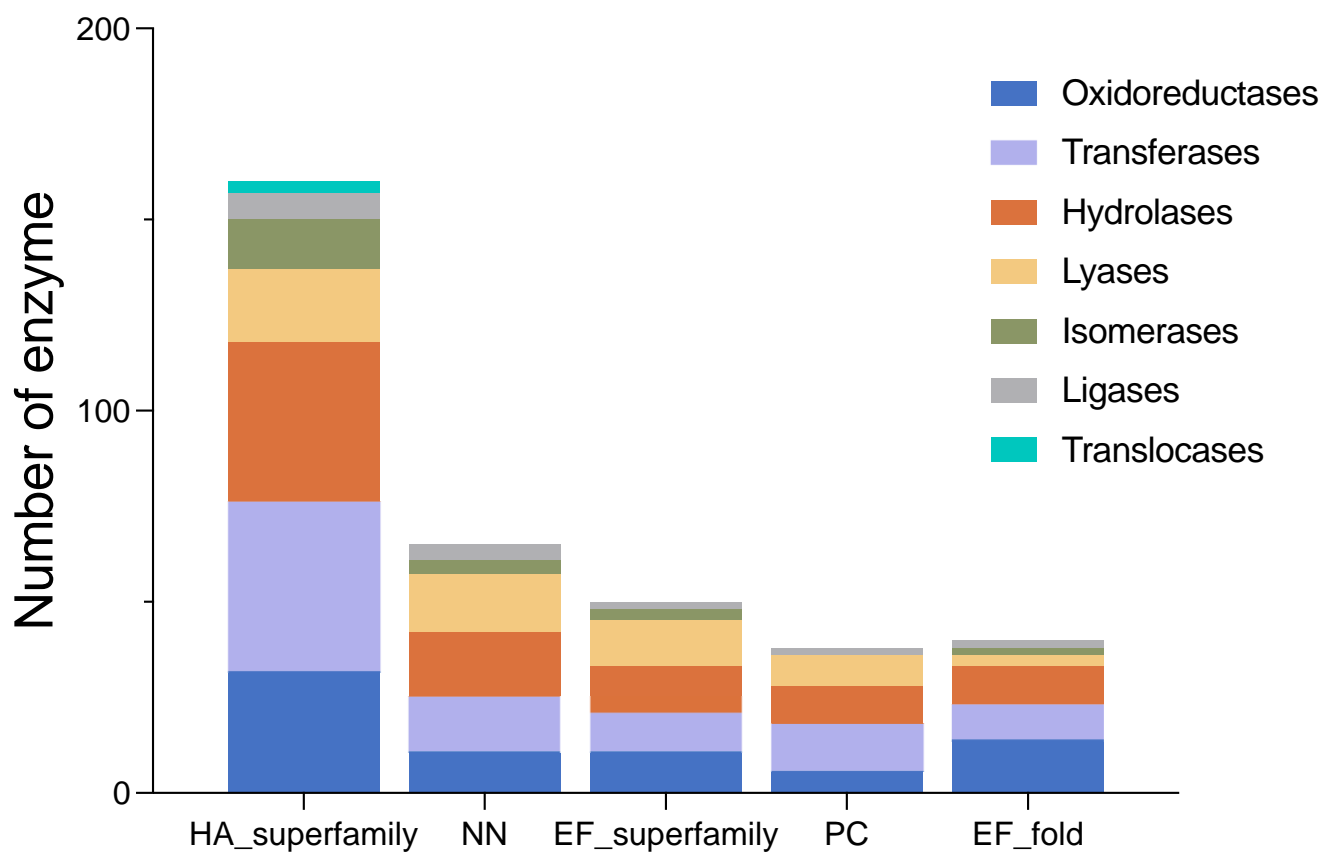

Supplement: qzae094_Supplementary_Data [file qzae094_supplementary_data.zip › Figure S1.pdf]

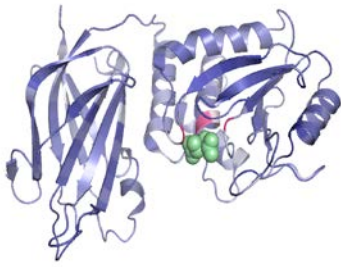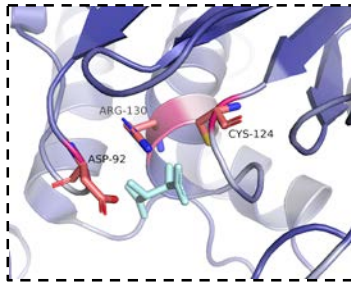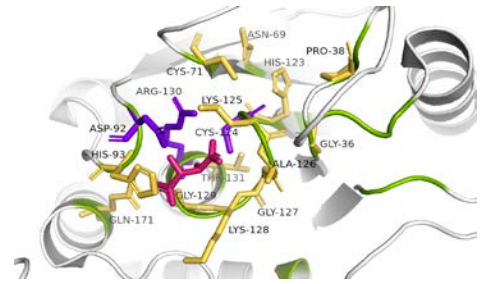

Supplement: qzae094_Supplementary_Data [file qzae094_supplementary_data.zip › Figure S13.pdf]

**NN**

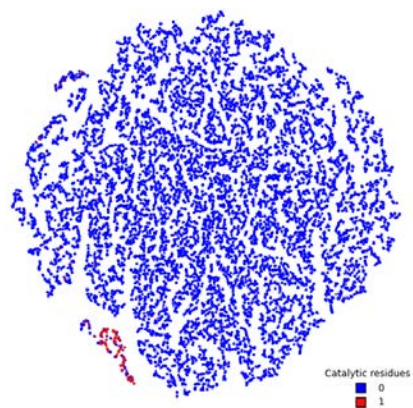

**HA\_superfamily**

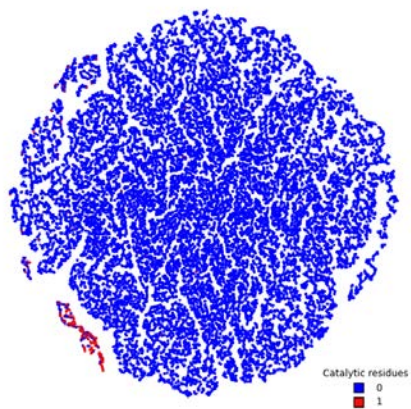

**EF\_superfamily**

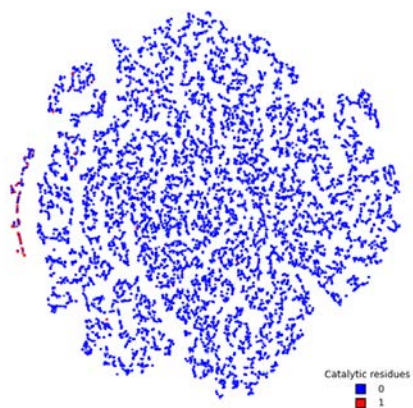

**EF\_fold**

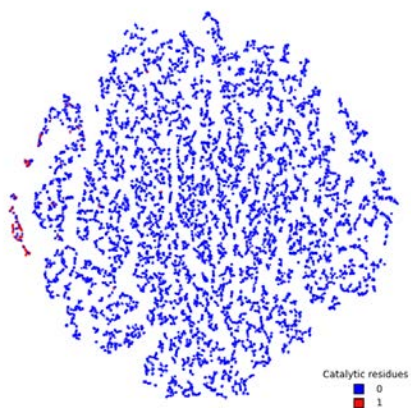

**PC**

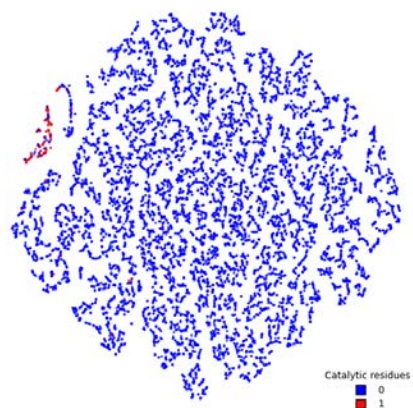

Supplement: qzae094_Supplementary_Data [file qzae094_supplementary_data.zip › Figure S11.pdf]

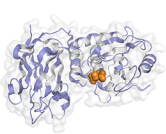

PTEN

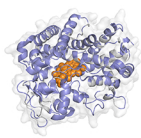

CYP2C9

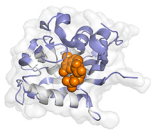

NUDT15

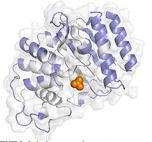

TEM1 beta-lactamase

Supplement: qzae094_Supplementary_Data [file qzae094_supplementary_data.zip › Figure S15.pdf]

**NN**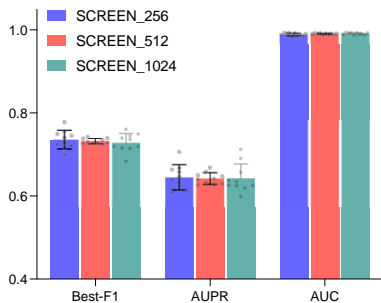**HA superfamily**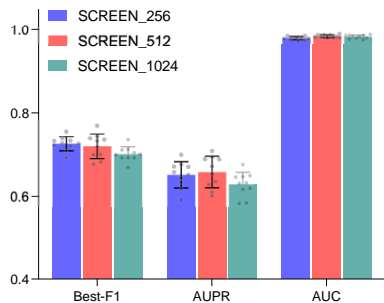**EFsuperfamily**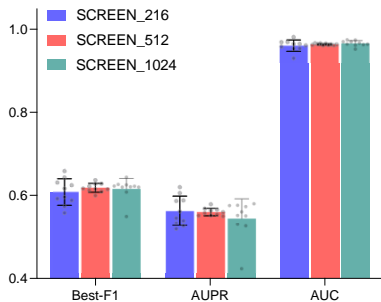**PC**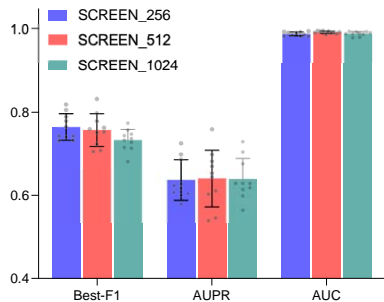**EFfold**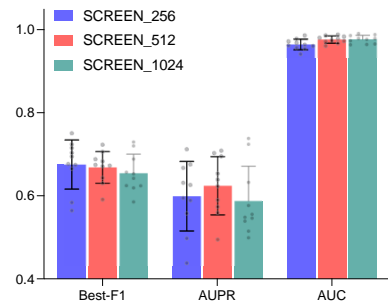

Supplement: qzae094_Supplementary_Data [file qzae094_supplementary_data.zip › Figure S4.pdf]

# Enzymes with different primary EC numbers

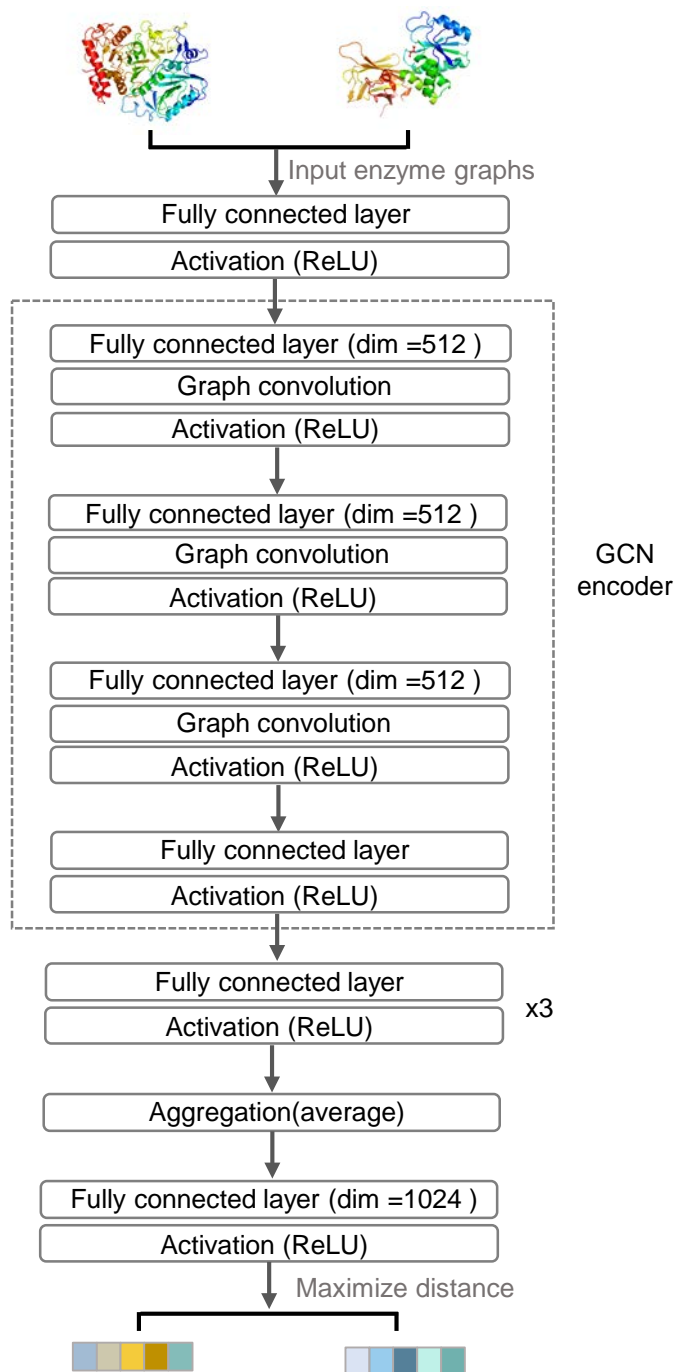

Supplement: qzae094_Supplementary_Data [file qzae094_supplementary_data.zip › Figure S2.pdf]

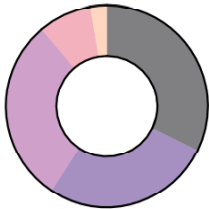

**Total=152**

- EF Superfamily dataset
- EF fold dataset
- HA superfamily dataset
- NN dataset
- PC dataset

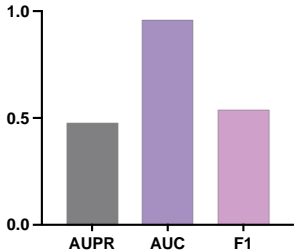

Supplement: qzae094_Supplementary_Data [file qzae094_supplementary_data.zip › Figure S9.pdf]

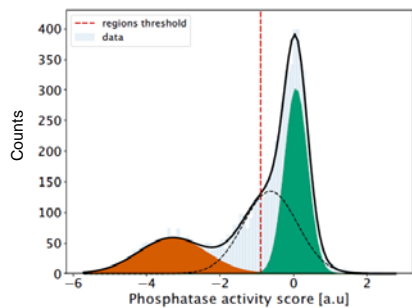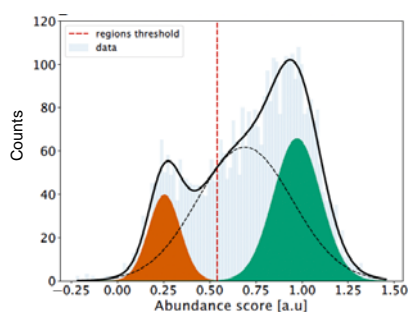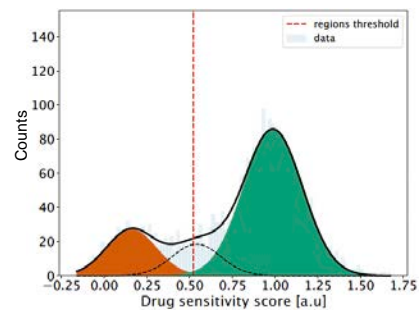

PTEN

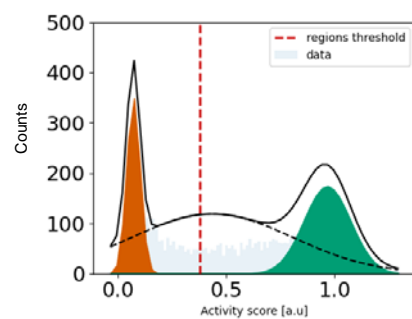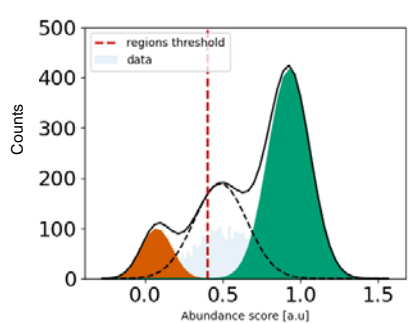

CYP2C9

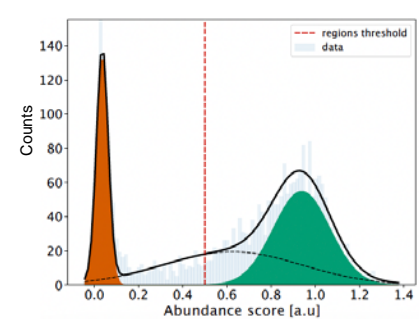

NUDT15

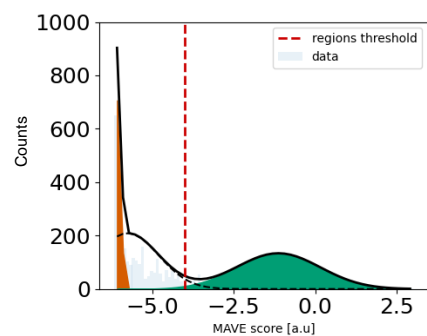

TEM1 beta-lactamase\_ECOLX

Supplement: qzae094_Supplementary_Data [file qzae094_supplementary_data.zip › Figure S14.pdf]

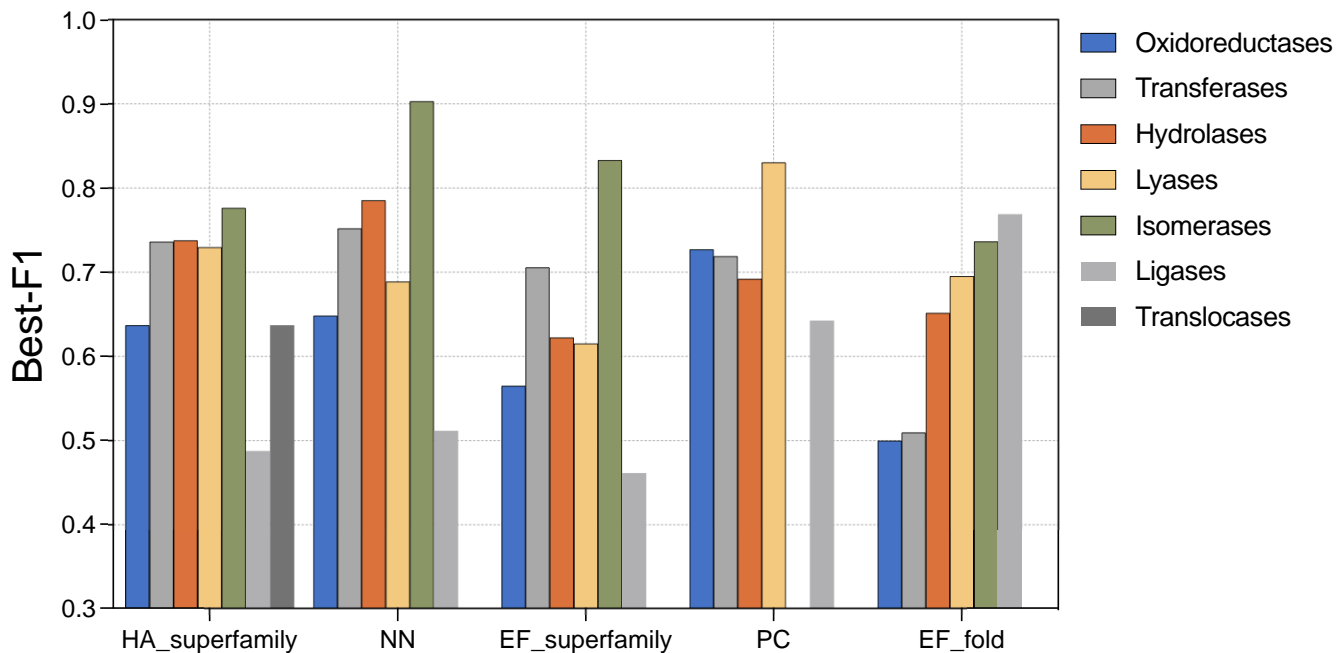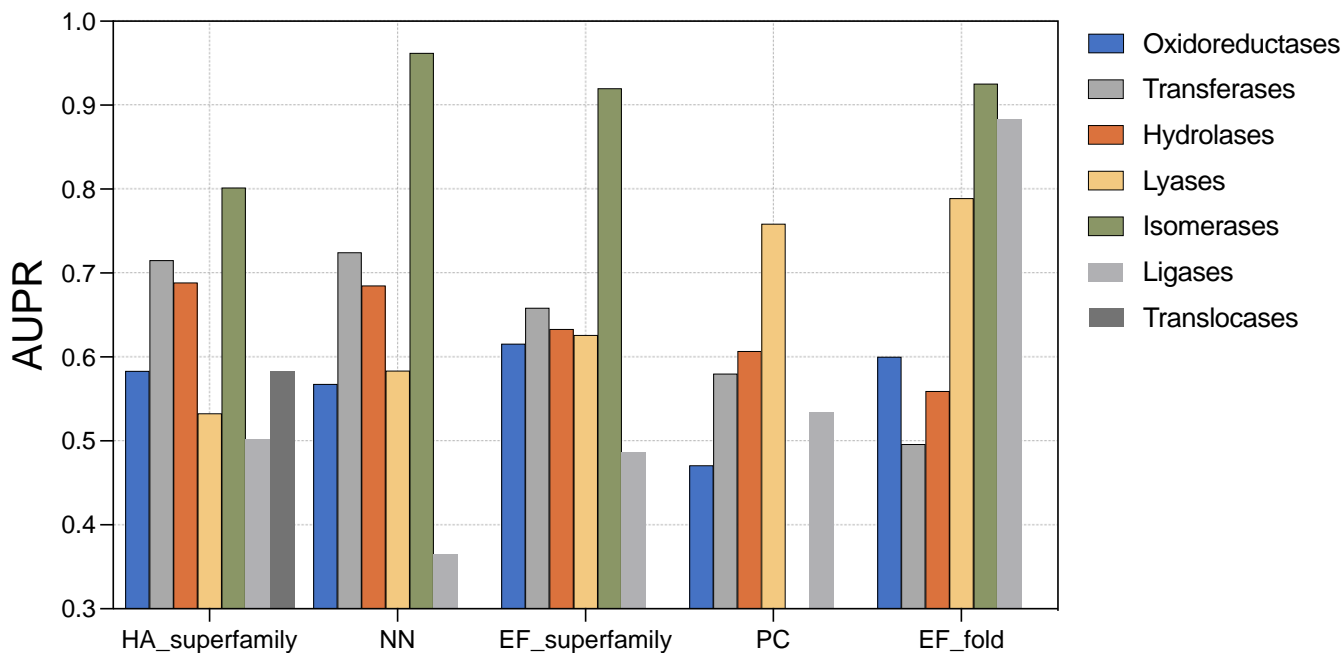

Supplement: qzae094_Supplementary_Data [file qzae094_supplementary_data.zip › Figure S6.pdf]
